# Plaque associated microglia hyper-secrete extracellular vesicles and accelerate tau propagation in a humanized APP mouse model

**DOI:** 10.1186/s13024-021-00440-9

**Published:** 2021-03-22

**Authors:** Kevin Clayton, Jean Christophe Delpech, Shawn Herron, Naotoshi Iwahara, Maria Ericsson, Takashi Saito, Takaomi C. Saido, Seiko Ikezu, Tsuneya Ikezu

**Affiliations:** 1 Departments of Pharmacology and Experimental Therapeutics, Boston University School of Medicine, Boston, MA 02118 USA; 2 Department of Cell Biology, Harvard Medical School, Boston, MA 02115 USA; 3 Department of Neurocognitive Science, Institute of Brain Science, Nagoya City University Graduate School of Medical Sciences, Nagoya, Aichi Japan; 4 Laboratory for Proteolytic Neuroscience, RIKEN Center for Brain Science, Wako, Saitama, Japan; 5 Center for Systems Neuroscience, Boston University, Boston, MA 02215 USA; 6 Department of Neuroscience, Mayo Clinic Florida, Jacksonville, FL 32224 USA

**Keywords:** Adeno-associated virus, Alzheimer’s disease, Amyloid-beta peptide, Amyloid precursor protein, Extracellular vesicles, Humanized mouse model, Lentivirus, Microglia, Microtubule-associated protein tau, Neuritic plaque, Tauopathy

## Abstract

**Background:**

Recent studies suggest that microglia contribute to tau pathology progression in Alzheimer’s disease. Amyloid plaque accumulation transforms microglia, the primary innate immune cells in the brain, into neurodegenerative microglia (MGnD), which exhibit enhanced phagocytosis of plaques, apoptotic neurons and dystrophic neurites containing aggregated and phosphorylated tau (p-tau). It remains unclear how microglia promote disease progression while actively phagocytosing pathological proteins, therefore ameliorating pathology.

**Methods:**

Adeno-associated virus expressing P301L tau mutant (AAV-P301L-tau) was stereotaxically injected into the medial entorhinal cortex (MEC) in C57BL/6 (WT) and humanized *APP* mutant knock-in homozygote (*App*^*NL-G-F*^) mice at 5 months of age. Mice were fed either chow containing a colony stimulating factor-1 receptor inhibitor (PLX5622) or control chow from 4 to 6 months of age to test the effect of microglia depletion. Animals were tested at 6 months of age for immunofluorescence, biochemistry, and FACS of microglia. In order to monitor microglial extracellular vesicle secretion in vivo, a novel lentiviral EV reporter system was engineered to express mEmerald-CD9 (mE-CD9) specifically in microglia, which was injected into the same region of MEC.

**Results:**

Expressing P301L tau mutant in the MEC induced tau propagation to the granule cell layer of the hippocampal dentate gyrus, which was significantly exacerbated in *App*^*NL-G-F*^ mice compared to WT control mice. Administration of PLX5622 depleted nearly all microglia in mouse brains and dramatically reduced propagation of p-tau in WT and to a greater extent in *App*^*NL-G-F*^ mice, although it increased plaque burden and plaque-associated p-tau^+^ dystrophic neurites. Plaque-associated MGnD microglia strongly expressed an EV marker, tumor susceptibility gene 101, indicative of heightened synthesis of EVs. Intracortical injection of mE-CD9 lentivirus successfully induced microglia-specific expression of mE-CD9^+^ EV particles, which were significantly enhanced in Mac2^+^ MGnD microglia compared to Mac2^−^ homeostatic microglia. Finally, consecutive intracortical injection of mE-CD9 lentivirus and AAV-P301L-tau into *App*^*NL-G-F*^ mice revealed encapsulation of p-tau in microglia-specific mE-CD9^+^ EVs as determined by super-resolution microscopy and immuno-electron microscopy.

**Discussion:**

Our findings suggest that MGnD microglia hyper-secrete p-tau^+^ EVs while compacting Aβ plaques and clearing NP tau, which we propose as a novel mechanistic link between amyloid plaque deposition and exacerbation of tau propagation in *App*^*NL-G-F*^ mice.

**Supplementary Information:**

The online version contains supplementary material available at 10.1186/s13024-021-00440-9.

## Background

Microglia are the immune cells of the central nervous system and possess a number of specific roles including synaptic pruning [1, 2], release of pro-inflammatory and anti-inflammatory cytokines [3, 4], as well as surveying for and phagocytosing pathologic insults [5–7]. Recently, a class of disease-activated microglia common in neurodegeneration called “MGnD” has been characterized along with their role in the alleviation or exacerbation of neurodegenerative disorders [8, 9]. MGnD exhibit characteristics similar to activated microglia and possess a unique molecular signature that is regulated by Triggering Receptor Expressed on Myeloid cells 2 (TREM2) and Apolipoprotein E (ApoE), which disrupts maintenance of central nervous system (CNS) homeostasis [8, 9]. MGnD microglia, which are identified via immunofluorescence against markers such as C-type lectin domain family 7, member A (Clec7A) and galectin-3 (Mac2), typically reside around amyloid plaques and phagocytose not only aggregated proteins, but also apoptotic neurons and synapses. There is still ongoing discussion as to whether MGnD microglia are ultimately beneficial or harmful in neurodegenerative disease. MGnD microglia may play a key role bridging amyloid plaque toxicity and tau pathology development in Alzheimer’s disease (AD). Amyloid plaques precede tau pathology in AD and are believed to initiate or build upon mechanisms responsible for tau pathology. Indeed, previous reports showed that amyloid-beta (Aβ) pathology accelerates tau pathology development in different mouse models [10–12]. Lately, the interplay between the spread of pathologic hyperphosphorylated tau (p-tau) and Aβ plaques has been a subject of investigation as p-tau^+^ aggregates deposited on plaques, referred to as “NP tau”, appear to affect sequestration and spread of pathologic tau seeds in a manner that may be dependent on microglia [10, 13]. It is still undetermined whether microglia play a critical role for the acceleration of tau propagation in the presence of Aβ plaque deposition.

One of the methods in which we can assess the effect of microglia on AD pathology is through their selective depletion. This is accomplished in mouse models of conditional knockdown of microglia, targeted depletion, or via administration of Colony stimulating factor 1 receptor (CSF1R) inhibitors [14–18]. By using CSF1R inhibitors, along with other means of selective microglia depletion, researchers examined the effect of depletion on amyloid plaque deposition, tau pathology and spread, synaptic integrity, as well as cognition in a variety of mouse models of neurodegeneration [19–23]. We previously established a rapid tau propagation model in which AAV-P301L-tau is injected into the medial entorhinal cortex (MEC) where p-tau is expressed and eventually propagated to the granular cell layer (GCL) of the hippocampal dentate gyrus (DG) in a manner facilitated by microglia and exosomes, which are small extracellular vesicles (EVs) synthesized in multivesicular bodies [22]. In the present study, we investigated how microglia may facilitate tau propagation by injecting AAV-P301L-tau into the MEC of C57BL/6 (WT) and *App*^*NL-G-F*^ knock-in mice while treating them with a CSF1R inhibitor. *App*^*NL-G-F*^ mice develop robust amyloid plaque formation by endogenously expressing three APP mutations [24].

In this study, we revealed that amyloid burden accelerated tau propagation in *App*^*NL-G-F*^ mice compared to WT mice and depleting microglia dramatically reduced tau propagation to the GCL. Additionally, increased deposition of NP tau as well as amyloid plaques following microglia depletion in *App*^*NL-G-F*^ mice suggested active clearance of protein aggregates by MGnD microglia. Interestingly, we observed that Clec7A^+^ MGnD microglia, activated in response to amyloid plaques and p-tau strongly expressed an exosomal marker, Tumor susceptibility gene 101, which was absent after microglia depletion. We further constructed a novel lentivirus expressing mEmerald-CD9 fusion protein (mE-CD9) in a microglia-specific manner and quantified the release of mE-CD9^+^ EV particles from single cells in vivo in the MEC. We found that the degree of EV release was over three times highe from Mac2^+^ MGnD microglia compared to Mac2^−^ microglia. Additionally, co-injection of mE-CD9 and AAV-P301L-tau revealed incorporation of p-tau within microglia-specific EVs. MGnD microglia, which are more prevalent in *App*^*NL-G-F*^, appeared to release 3–5 times more p-tau through EVs compared to homeostatic microglia. These data highlight a possible mechanism for the accelerated tau propagation witnessed in *App*^*NL-G-F*^ mice via MGnD microglia-derived EVs and may shed light on the conflicting microglial roles in AD pathology development and progression.

## Results

### Microglia depletion increases plaque deposition

**Fig. 1:**
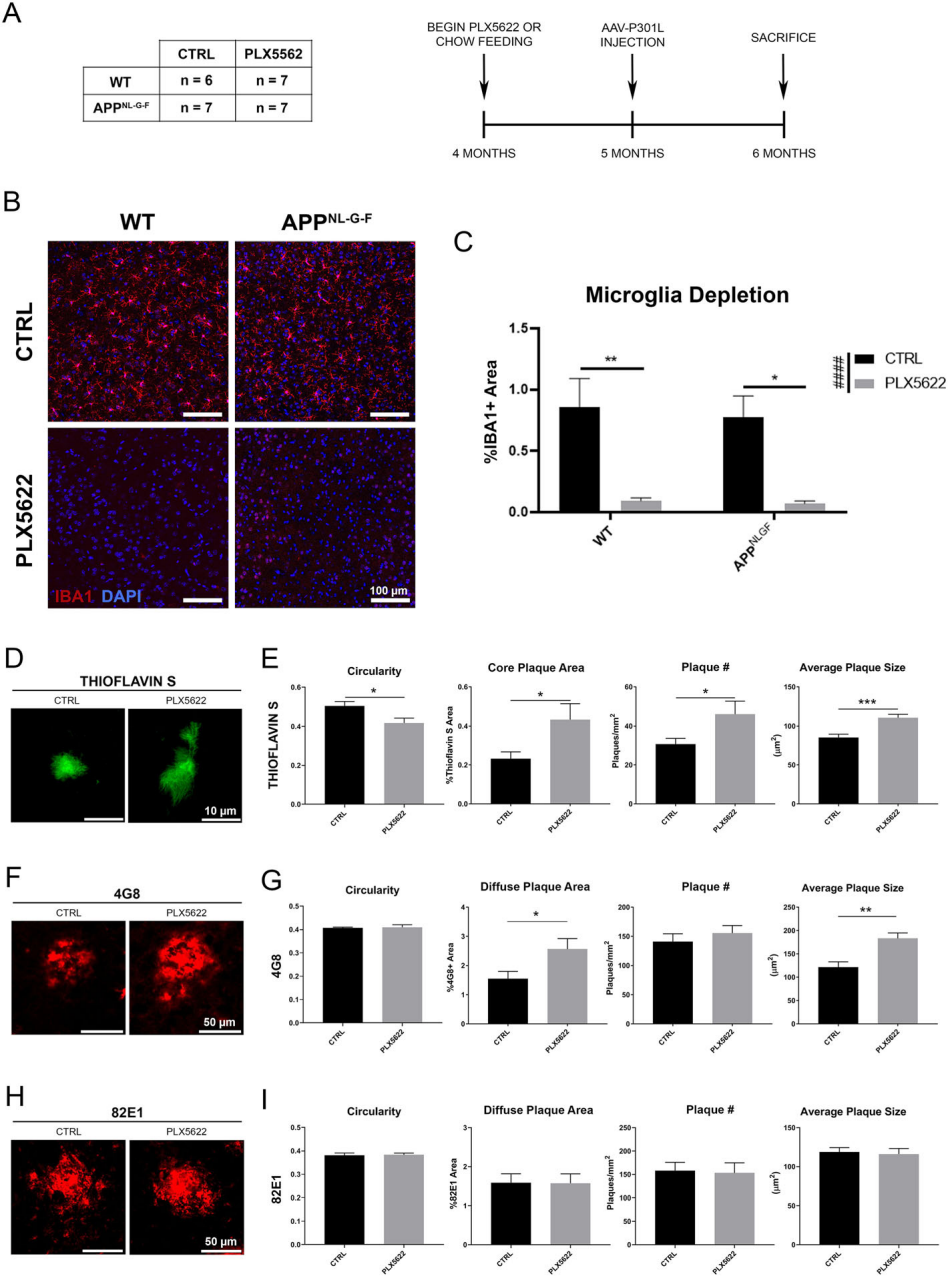
PLX5622 treatment ablates microglia and increases amyloid plaque burden. **a.** Schematic of study design with PLX5622 administration. The table displays the number of mice used per experimental group. **b.** Representative images of IBA1 staining in the cortical region from WT and *App*^*NL-G-F*^ mice administered with control (CTRL) or PLX5622 chow. **c.** Unbiased quantification of percentage IBA1^+^ area in the cortex. **d.** Representative images of Thioflavin-S staining in the cortex. **e.** Unbiased quantification of plaque characteristics in Thioflavin-S stained slices in the cortex. **f.** Representative images of 4G8 staining in the cortex. **g.** Unbiased quantification of plaque characteristics in 4G8 stained slices in the cortex. **h.** Representative images of 82E1 staining in the cortex. **i.** Unbiased quantification of the plaque characteristics in 82E1 stained slices in the cortex. Representative images displayed in **a-i** are a mix of male and female mice. All values displayed in **a-i** represent the mean ± standard error (SEM) for a minimum of 6 animals per group. Graphs comparing values across all 4 groups were analyzed via 2-way analysis of variance (ANOVA) with Tukey post-hoc analysis for individual comparisons. Graphs comparing two groups were analyzed via Unpaired t-test. ^*^p < 0.05, ^**^ *p* < 0.01, ^***^ *p* < 0.001, between indicated groups. #### *p* < 0.0001 for the PLX5622 factor

Immunofluorescence against IBA1, a marker for microglia and macrophages [25], showed that IBA1 was almost completely absent (> 93%) after 2 months of drug treatment in both groups (Fig. 1a-c). Additionally, overall P2RY12^+^ area is not statistically different between WT and *App*^*NL-G-F*^ mice, which is also depleted by PLX5622 (Fig. S1A-B). In the AD brain, microglia become activated in response to amyloid pathology and migrate to the region where they compact and phagocytose Aβ plaques [26–29]. We hypothesized that microglia depletion altered the Aβ plaque compaction in *App*^*NL-G-F*^ mice. Staining with thioflavin-S revealed the circularity of dense-core plaques was significantly reduced following microglia depletion, suggesting that Aβ plaques were less compacted in the absence of microglia (Fig. 1d-e). Consistent with this data, overall Aβ plaque area, number, and size were significantly increased by microglia depletion (Fig. 1e). 3D surface renderings of thioflavin-S plaques revealed a dramatic decrease in sphericity and increase of plaque volume and area (Fig. S1C-D). These findings were mostly reproduced in diffuse amyloid staining with 4G8 (detecting Aβ17–24, Fig. 1f-g), specifically in the size of plaques and overall plaque area. Interestingly, immunofluorescence for diffuse amyloid with an alternative antibody, 82E1, revealed no differences in plaque pathology after microglia depletion in the same brains (Fig. 1h-i). Moreover, 82E1 possessed a distinct staining pattern from 4G8 (Fig. S1E). The 82E1 antibody detects Aβ1–16, which is analogous to the 6E10 antibody, most commonly used in studies assessing the effect of microglia depletion on amyloid burden (Supplementary Table S1). The discrepancy in the Aβ plaque detection between 82E1 and 4G8 antibodies may be an important consideration for future studies. In addition, immunofluorescence against the astrocytic marker, Glial fibrillary acidic protein (GFAP) revealed that microglia depletion had no impact on astrogliosis in WT mice (Fig. S1F-G), confirming results from a previous report [30]. Furthermore, there was a robust increase in astrogliosis around Aβ plaques in *App*^*NL-G-F*^ mice compared to WT mice that was unaffected by PLX5622 treatment (Fig. S1F-G). Similarly, a previous report indicated that microglia depletion did not affect astrogliosis in response to tau pathology [23]. Together, these results strongly suggest that microglia play a significant role in prevalence of Aβ plaques as well as their compaction in *App*^*NL-G-F*^ mice between 4 to 6 months of age.

### Enhanced tau propagation and its reduction by CSF1R inhibition in *App*^*NL-G-F*^ mice

**Fig. 2:**
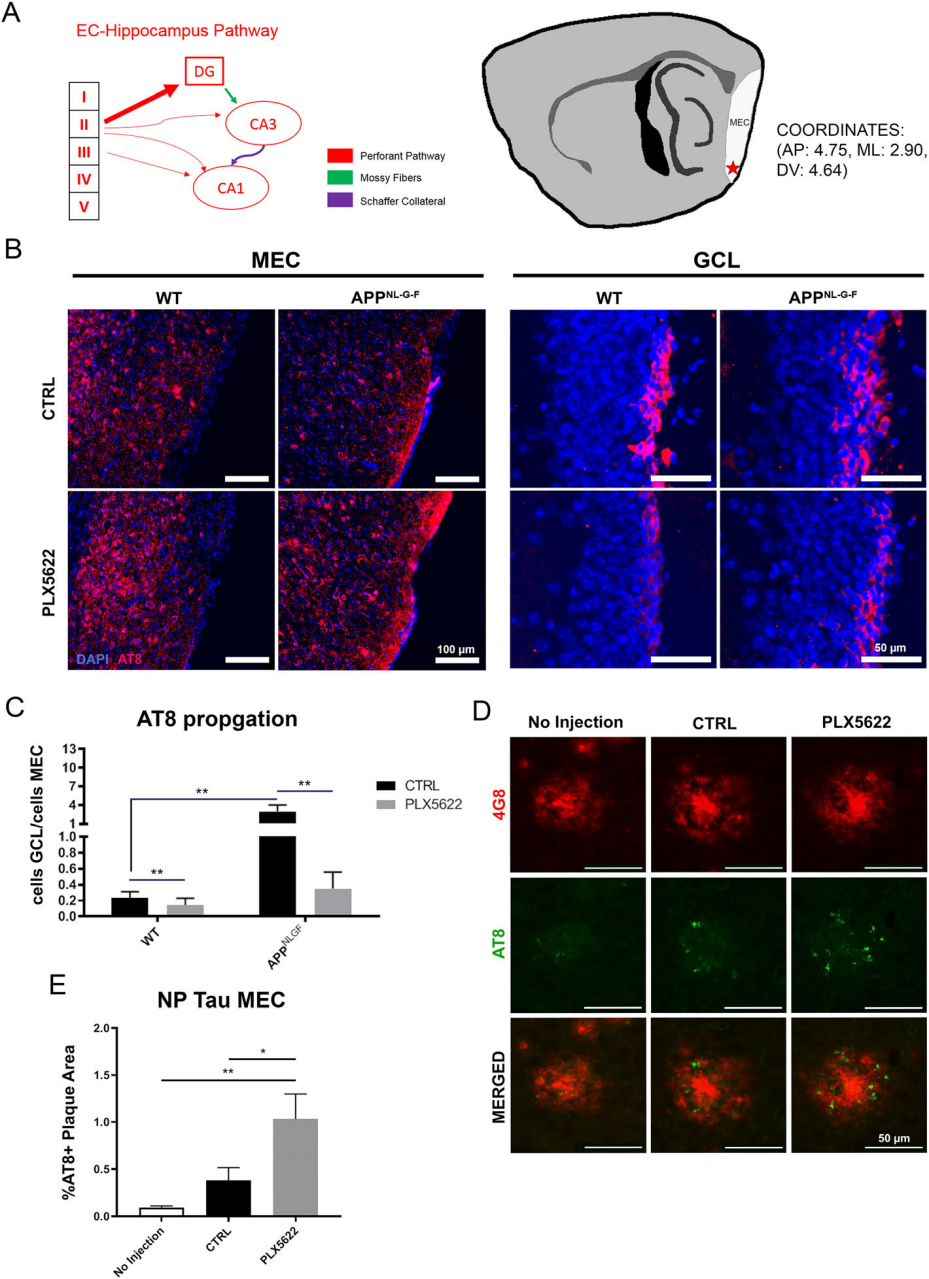
Aβ deposition accelerates tau propagation in a microglia-dependent manner, while microglial depletion enhances NP tau formation. **a.** Schematic of injection coordinate and tri-synaptic pathway. **b.** Representative images of AT8 staining in the MEC and the GCL region of the DG. **c.** Unbiased quantification of AT8^+^ cell propagation from the MEC to the GCL. Tau propagation values across all 4 treatment groups are displayed. **d.** Representative image of plaques in the MEC of non-injected *App*^*NL-G-F*^ mice, injected mice receiving control chow, and injected mice receiving PLX5622. **e.** Unbiased quantification of percentage AT8^+^ plaque area within the MEC. Representative images displayed in **a-e** are a mix of male and female mice. All values displayed in **a-e** represent the mean ± SEM. Graphs comparing values across 4 groups were analyzed via 2-way ANOVA with Tukey post-hoc analysis for individual comparisons. Graphs comparing values across 3 groups were analyzed via 1-way ANOVA with Fisher’s LSD post-hoc analysis for individual comparisons. ^*^ *p* < 0.05, ^**^ *p* < 0.01 between indicated groups

P-tau propagates from the MEC to the hippocampus, which are anatomically-connected regions as seen in the process of tau pathology development in AD. The perforant pathway projects from the MEC to the DG and composes the tri-synaptic circuit together with mossy fibers connecting to Cornu Ammonis 3 (CA3) and the Schaffer collaterals to CA1 (Fig. 2a) [31]. To determine the effect of microglia depletion on tau propagation via the perforant pathway in *App*^*NL-G-F*^ mice, animals were treated 1 month with PLX5622 or control chow from 4 months of age. AAV2/6 pseudotyped synapsin-1 promoter-driven transgene expression of P301L MAPT mutant (AAV-P301L-tau) was injected into the MEC (AP: 4.75, ML: 2.90, DV: 4.64) in *App*^*NL-G-F*^ and WT mice at 5 months of age as previously described (Fig. 2a) [22]. The animals were fed PLX5622 or control chow for another month until the end point of the study (2 months treatment in total). Tau propagation from the MEC to the DG was assessed by the immunofluorescence against p-tau (AT8, detecting pSer^202^/pSer^205^ tau). Following injection of AAV-P301L-tau, we observed strong AT8^+^ cell soma staining in the MEC and in the GCL of the DG in *App*^*NL-G-F*^ mice and also in WT mice to a lesser extent (Fig. 2b). Furthermore, we also found AT8^+^ cells in the hilus and CA1 regions in both WT and *App*^*NL-G-F*^ mice (Fig. S2A-B), suggesting that p-tau was propagated through not only the perforant pathway, but also possibly the temporoammonic pathway, which projects from the EC layer II to CA1 [32]. These results were consistent with previous reports showing an augmented effect of amyloid plaques on tau propagation [10, 12, 33]. Strikingly, microglia depletion effectively suppressed the tau propagation in both groups (Fig. 2c), and notably the inhibitory effect of microglia depletion on tau propagation was more prominent in *App*^*NL-G-F*^ compared to WT mice.

In addition to AT8^+^ neurons, neuritic plaque-associated AT8^+^ tau deposits (NP tau) were investigated. One recent study demonstrated the significant effect of TREM2 mutation on NP tau formation in tau fibril-injected APPPS1–21 mouse brains, suggesting microglial involvement in NP tau development [13]. We thus examined the effect of AAV-P301L-tau propagation on NP tau formation in *App*^*NL-G-F*^ mice with or without microglial depletion. Interestingly, AT8^+^ NP tau surrounding plaques was increased following AAV-P301L-tau incubation in the MEC, which was further significantly enhanced by microglia depletion (Fig. 2d-e). In the hippocampus, proportions of NP tau showed similar patterns following injection and microglia depletion, but were insignificantly different (Fig. S2C). This suggests that tau expressed in the MEC can also spread and be incorporated in NP tau, which may be actively phagocytosed by plaque-associated microglia. Together, these data provide a strong evidence that Aβ deposition exacerbates tau propagation in a microglia dependent manner.

### Increased synaptic tagging and EV release signatures in the amyloid bearing brain

**Fig. 3:**
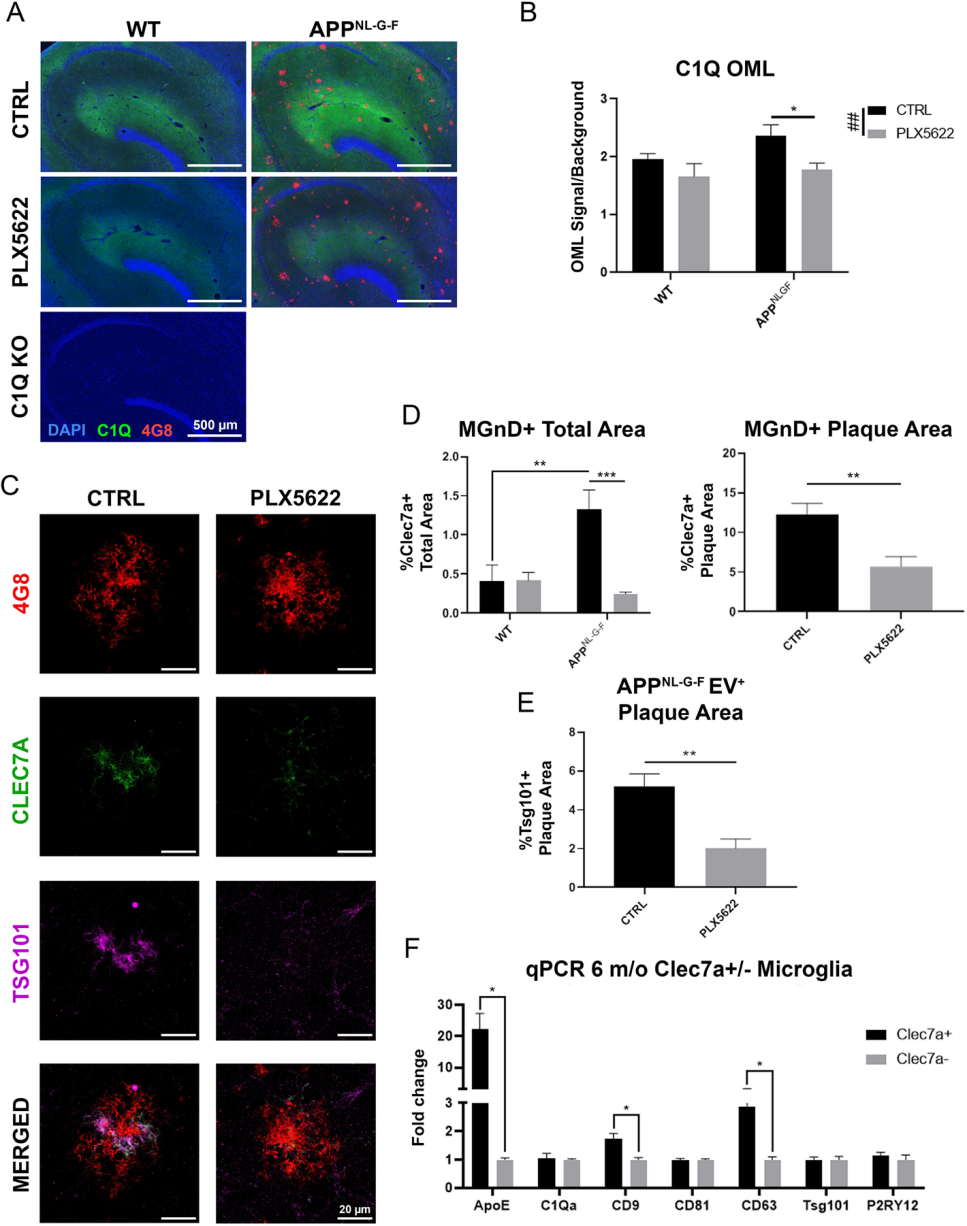
Increased synaptic tagging and EV release signatures in the amyloid bearing brain. **a.** Representative images of C1Q, 4G8, and DAPI staining in the hippocampal region of WT and *App*^*NL-G-F*^ mice administered with control (CTRL) or PLX5622 chow as well as C1Q KO mice. **b.** Unbiased quantification of C1Q intensity in the OML of the hippocampus. **c.** Representative stacked confocal images of 4G8, Clec7A, and Tsg101 staining of plaques in the OML. See also Supplemental Fig. S3A and Video S1. **d.** Unbiased quantification of percentage Clec7A^+^ area across the OML for all groups and of the plaque-specific regions. **e.** Unbiased quantification of Tsg101 intensity of plaque-positive regions in the OML compared to background signal. **f.** qPCR data displaying fold-increases in MGnD and EV-associated markers ApoE, C1Qa, CD9, CD81, CD63, Tsg101, and P2RY12 expressed in Clec7A^+^ over Clec7A^−^ microglia. Representative images displayed in **a-f** are a mix of male and female mice. All values displayed in **a-f** represent the mean ± SEM for a minimum of 3 animals per group. Graphs comparing values across all 4 groups were analyzed via 2-way ANOVA with Tukey post-hoc analysis for individual comparisons. Graphs comparing two groups were analyzed via Unpaired t-test. ^*^ *p* < 0.05, ^**^ *p* < 0.01, and ^***^ *p* < 0.001 between indicated groups. ^##^ *p* < 0.01 for the PLX5622 factor


C1q is a protein complex known to be a part of the complement system and is also mostly derived from microglia [34]. It is highly deposited on synapses in the molecular layer of the DG [35, 36]. The expression level of C1q in the outer molecular layer was significantly reduced in *App*^*NL-G-F*^ mice following microglia depletion, revealing the absence of microglia-derived proteins (Fig. 3a-b). There was no significant change in C1q positivity in WT mice by microglia depletion and C1q knockout mice showed no positivity as a negative control. In the AD brain, microglia become activated in response to amyloid pathology and migrate to compact and phagocytose plaque material [26–29]. We next examined MGnD microglia in the OML and specifically around plaque regions in *App*^*NL-G-F*^ mouse brains via Clec7A staining [8]. Clec7A^+^ MGnD microglia were detected concentrated around plaques and were ~ 80% eliminated following PLX5622 treatment in *App*^*NL-G-F*^ mice (Fig. 3c-d). Microglia are known to produce and secrete EVs more efficiently following activation [37]. We found striking co-expression of Tumor susceptibility gene 101 (Tsg101), an EV marker in Clec7A^+^ MGnD microglia, which was also diminished by PLX5622 treatment (Fig. 3c, e, Supplemental Video S1). This suggests that MGnD microglia may be over-synthesizing EVs. Homeostatic microglia, indicated by IBA1^+^/Clec7A^−^ cells, do not colocalize with Tsg101 (Fig. S3A). Additionally, Clec7A^+/−^ microglia were isolated from 6-month-old *App*^*NL-G-F*^ mice via fluorescence-activated cell sorting (FACS) via staining for CD11b, FCLRS, and Clec7A (Fig. S3B). Clec7A^+^ microglia show significantly upregulated gene expression of EV markers, such as *Cd9* and *Cd63*, along with MGnD marker *ApoE* compared to Clec7A^−^ microglia (Fig. 3f). Additionally, expression of P2RY12 was not affected by the MGnD phenotype. Lastly, plaque-associated Clec7A^+^ MGnD microglia appear to internalize NP tau (Fig. S3C and Video S2), which supports our hypothesis that MGnD microglia may actively phagocytose NP tau and dystrophic neurites near the plaques. Together, these findings suggest that MGnD microglia phagocytose NP tau and hyper-secrete EVs, which may influence the spread of pathologic tau seeds.

**Additional file 2.** **Supplementary Video S1.** Phagocytosis of NP tau by Clec7a+ microglia.


**Additional file 3.** **Supplementary Video S2.** Co-localization of Tsg101 in Clec7a+ microglia.

**Fig. 4:**
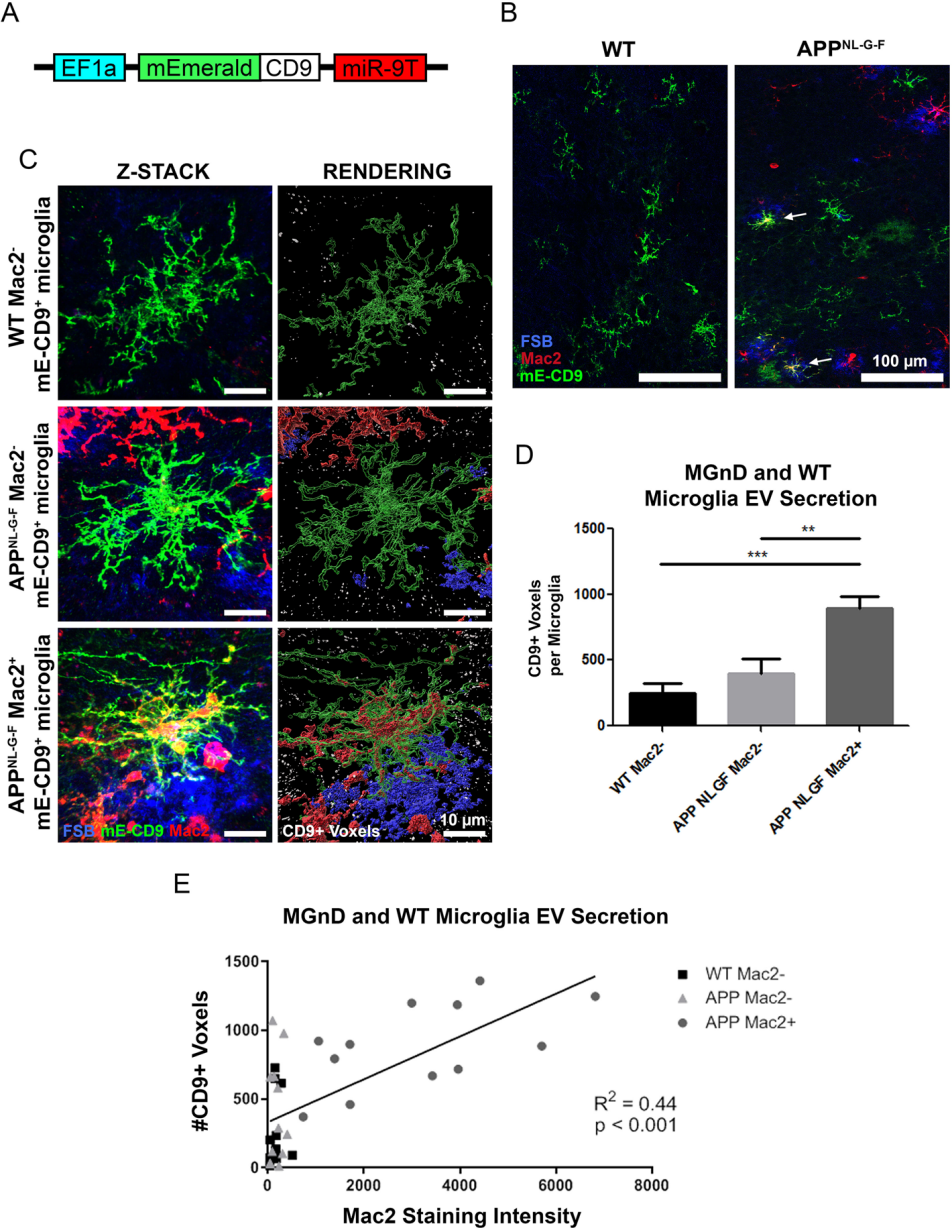
In vivo imaging of EV secretion from microglia after lentiviral microglia-specific expression of mE-CD9. **a.** Schematic of mE-CD9 lentiviral construct. **b.** Representative images of mE-CD9 lentivirus transduction in WT and *App*^*NL-G-F*^ microglia following injection and 10-day incubation. **c.** Representative images of individual microglia and released mE-CD9^+^ particles showing FSB, Mac2, and mE-CD9. **d.** Unbiased quantification of mE-CD9^+^ voxels surrounding individual microglia (*n* = 12 microglia per group from 3 mice per group). **e.** Regression plot of Mac2 staining intensity versus the number of mE-CD9 particles released by individual microglia. Representative images displayed in **a-e** are a mix of male and female mice. All values displayed in **a-e** represent the mean ± SEM for a minimum of 3 animals per group. Graphs comparing values across all 4 groups were analyzed via 2-way ANOVA with Tukey post-hoc analysis for individual comparisons. Graphs comparing two groups were analyzed via unpaired t-test. ^**^ *p* < 0.01, ^***^ *p* < 0.001, between indicated groups

To directly determine if MGnD microglia release more EVs compared to homeostatic microglia in vivo, we developed a novel microglia-specific lentivirus expressing mEmerald-CD9 (mE-CD9) fusion protein (Fig. 4a) [38]. This lentivirus contains the tandem miR-9 target sequence (miR9T) on the 3’UTR, which is targeted by miR-9-expressing neuronal cells and ensures silencing of gene expression in any non-microglial cells [39] and therefore achieving microglia-specific expression of mE-CD9. We also incorporated the EF1α promoter, which is highly active in murine microglia [40]. In vitro transduction of HEK293T cells demonstrates transgene expression within 48 h (Fig. S4A). mE-CD9 was enriched in the EV fraction of the conditioned media compared to the cell lysate (Fig. S4B). The high-titer (~ 1 × 10^9^ TU/mL) mE-CD9 lentivirus was bilaterally injected into the MEC of both WT and *App*^*NL-G-F*^ mice at 6 months of age. Following 10-day incubation, microglia-specific expression of mE-CD9 was detected by GFP antibody staining (Fig. 4b). MGnD microglia, which are indicated by Mac2 staining positivity, were observed as mE-CD9^+^ plaque-associated microglia in *App*^*NL-G-F*^ brains (indicated by white arrows), and were absent in WT brains lacking AAV-P301L-tau injection. Triple immunofluorescence staining of mE-CD9, IBA1 and P2RY12 showed that ~ 94% of mE-CD9^+^ signal co-localized with these microglia markers (Fig. S4C), indicating microglia-specific expression of mE-CD9. MGnD were detected by Mac2 staining [41], which coincide with Clec7A^+^ MGnD nearly 100% of the time (Fig. S4D), suggesting that both antibodies detect the same population of MGnD microglia. Z-stack images of mE-CD9^+^/Mac2^−^ microglia in WT mice, and mE-CD9^+^/Mac2^±^ microglia in *App*^*NL-G-F*^ mice were captured via Leica SP8 with Lightning super-resolution confocal microscope and processed in IMARIS rendering software to automatically quantify the number of mE-CD9^+^ EV particles (white voxels) surrounding individual microglia (Fig. 4c). Quantification of mE-CD9^+^ EV particles localized around individual microglia revealed that Mac2^+^ MGnD microglia released over 3 times as many EVs as Mac2^−^ microglia (Fig. 4d). The intensity of Mac2 staining in microglia showed a strong positive correlation with the particle number of EVs, suggesting that the MGnD phenotype positively influenced EV release (Fig. 4e). We then isolated the EVs from the left hemisphere of the bilaterally-injected brains using discontinuous sucrose gradient ultracentrifugation method as previously described [42, 43]. Biochemical quantification of mE-CD9 was performed using whole brain homogenate and extracellular vesicles isolated from mE-CD9 lentivirus-injected brains by GFP ELISA, which cross-react with mEmerald (Fig. S4E). There was significant enrichment of mE-CD9 in the EV fraction over brain homogenate both in WT and *App*^*NL-G-F*^ mice, biochemically confirming the secretion of mE-CD9^+^ EVs in vivo. Taken together, these data demonstrate that MGnD microglia, elicited here by Aβ plaques, secrete significantly more EVs than non-MGnD microglia in *App*^*NL-G-F*^ mice in vivo.

### P-tau is encapsulated within microglia-specific EVs, which is augmented in MGnD microglia

**Fig. 5:**
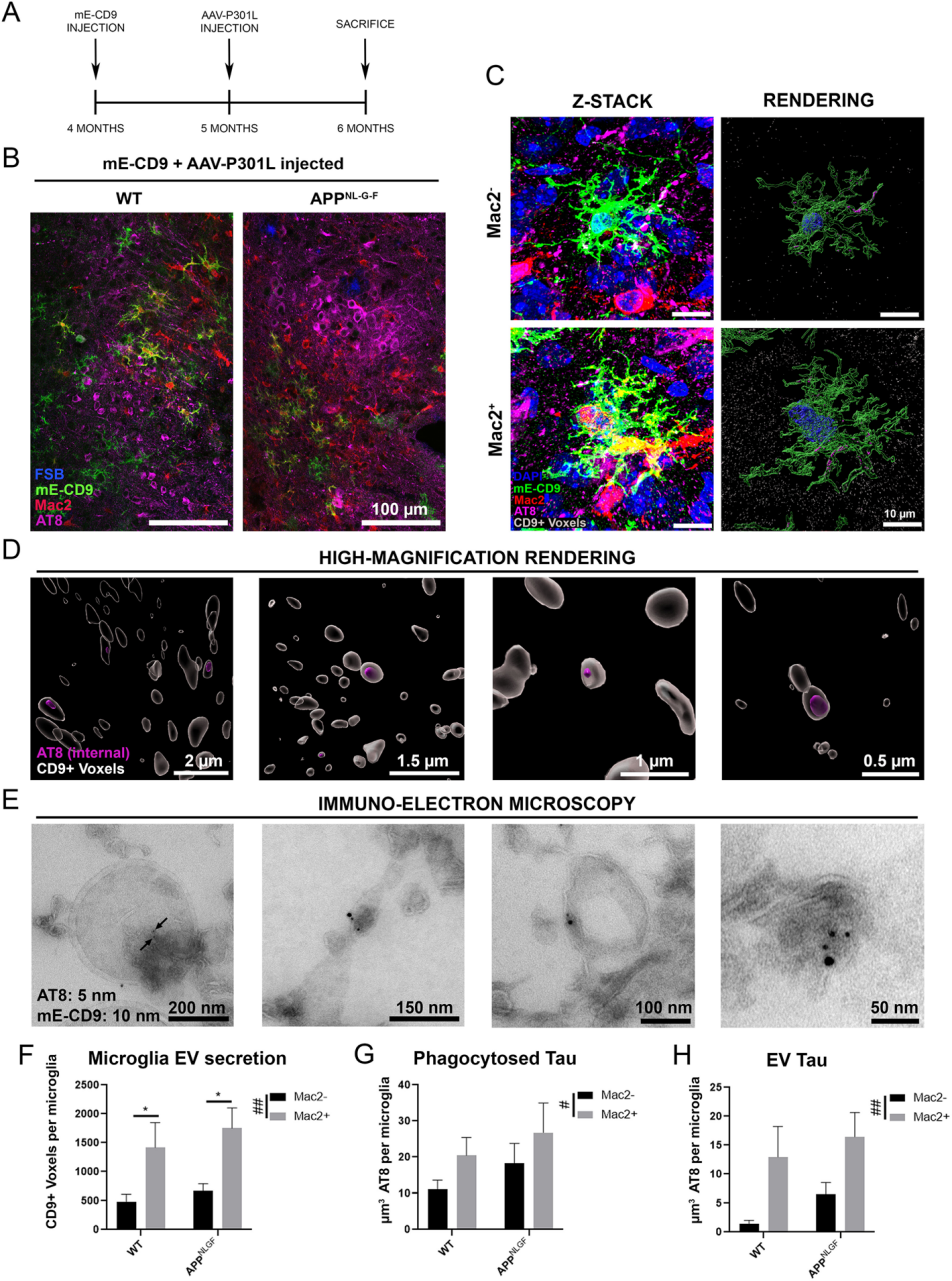
Pathologic tau is secreted through microglia-derived EVs. **a**: The timeline of injection of mE-CD9 lentivirus and AAV-P301L-tau. **b**: Representative low-magnification images of the injection site in the MEC showing FSB (blue: amyloid plaque), mE-CD9 (green), Mac2 (red), and AT8 (magenta). **c**: Left: Representative images of homeostatic microglia (Mac2^−^) and MGnD (Mac2^+^) with phagocytosed AT8^+^ p-tau and secreted mE-CD9^+^ EVs (white) surrounding microglia. Right: Surface rendering images by IMARIS software showing internalized p-tau within microglia or EVs. **d**: Representative images of microglia-derived mE-CD9^+^ EVs (white) containing p-tau (magenta). See also Supplementary Video S3. **e**: Immuno-gold electron microscopy images of microglia-derived EV containing mE-CD9 fusion protein (GFP^+^ 10 nm immuno-gold dots) and p-tau (AT8^+^ 5 nm immuno-gold dots). **f**: Quantification of mE-CD9^+^ EVs released from homeostatic microglia (Mac2^−^) and MGnD (Mac2^+^) in the injected region. **g**: Volume of phagocytosed p-tau per microglia **h**: Quantification of total p-tau released through mE-CD9^+^ EVs per microglia. Representative images displayed in **a-h** are a mix of male and female mice. **f-h**: *n* = 8 microglia per group from 5 WT and 5 *App*^*NL-G-F*^ animals. Graphs comparing values across all 4 groups were analyzed via 2-way analysis of variance (ANOVA) with Tukey post-hoc analysis for individual comparisons. * *p* < 0.05 between indicated groups, # *p* < 0.05, ## *p* < 0.01 for the Mac2 factor


If the hypersecretion of EVs by MGnD microglia is involved in the seeding and propagation of pathologic tau between brain regions, we should be able to observe p-tau encapsulated by microglia-derived EVs. In order to investigate this possibility, we bilaterally injected mE-CD9 lentivirus into the MEC of 4-month-old WT and *App*^*NL-G-F*^ mice followed by the injection of AAV-P301L-tau in the same region one month later (Fig. 5a). Mice were euthanized at 6 months of age. Immunofluorescence staining for GFP in order to detect mE-CD9 and AT8 for p-tau labeling indicated successful co-expression of mE-CD9 lentivirus and AAV-P301L-tau in the same region (Fig. 5b). MGnD microglia, indicated by Mac2^+^ microglia, were elicited in response to AAV-P301L-tau injection in both mice, but were significantly more prevalent in *App*^*NL-G-F*^ mice compared to WT control mice (Fig. S5A). Confocal z-stack images of individual p-tau associated microglia demonstrated that there were both homeostatic microglia and MGnD microglia in the p-tau-expressing region in either group (Fig. 5c). As seen before, these microglia appeared to be surrounded by mE-CD9^+^ EVs. Renderings of high-magnification images revealed that p-tau was encapsulated by microglia-specific EVs around homeostatic microglia and MGnD microglia in the tau-injected brain region (Fig. 5d and Video S3). EVs were isolated from co-injected brains as described before and subjected to double immuno-gold labeling of AT8 to detect p-tau (5 nm immuno-gold dot) and GFP to detect membrane-localized mE-CD9 (10 nm immuno-gold dot). Immuno-electron microscopic captured images of EVs revealed the presence of AT8^+^ p-tau encapsulated by GFP^+^ microglia-specific EVs (Fig. 5e). EVs surrounding Mac2^−^ and Mac2^+^ microglia were quantified as done previously (*n* = 8 microglia per group). MGnD microglia secreted roughly three times as many as EVs as homeostatic microglia (Fig. 5f). Additionally, the amount of p-tau internalized by these microglia and their EVs was quantified (Fig. 5g-h). Regardless of whether in WT or *App*^*NL-G-F*^ mice brains, Mac2^+^ MGnD microglia significantly internalized and secreted p-tau^+^ mE-CD9^+^ EVs more than Mac2^−^ homeostatic microglia. These data suggest that microglia-derived EVs distribute pathologic tau seeds, a phenomenon that is enhanced by the phenotypic shift of microglia into MGnD phenotype.

**Additional file 4.** **Supplementary Video S3.** p-tau+ EV secretion from microglia.

## Discussion

### Microglia depletion and tauopathy

The role of microglia in mediating the interplay between amyloid and tau pathologies remains elusive. In the current study, we demonstrated that abnormally phosphorylated and aggregated tau propagates from the MEC to the DG in WT and *App*^*NL-G-F*^ mice in a microglia-dependent manner. We further provided evidence that the neurodegenerative microglia phenotype MGnD produce and secrete abundant EVs containing p-tau, indicating a novel mechanism for how activated microglia may facilitate development and propagation of tau pathology. We previously reported the beneficial effects of microglia depletion as well as inhibition of EV synthesis and secretion in preventing tau spread in WT and P301S tau transgenic mice [22, 44]. We witnessed the same, but even more beneficial effect of microglia depletion in a mouse model exhibiting the other pathological hallmark of AD, Aβ plaques. Several studies including ours demonstrate the beneficial effect of CSF1R inhibitor-mediated microglia depletion on halting tauopathy in different tau mouse models [22, 30, 45]. This includes the AAV-mediated tau propagation system and P301S mice, but not in hTau mice expressing human wildtype tau or aged rTg4510 P301L tau mice, in which only partial microglial depletion was achieved (Supplementary Table S2) [23, 46]. One CSF1R inhibitor, JNJ-40346527, was successful in suppressing tauopathy development in P301S tau mice and is being tested in a Phase 1 study of AD cases (NCT04121208).

In this study, we utilized the *App*^*NL-G-F*^ mouse model and CSF1R inhibitor PLX5622 to determine the effect of MGnD microglia on tau pathology development. It was noted previously that at lower doses of PLX5622 treatment, plaque-associated MGnD microglia are specifically depleted whereas homeostatic microglia are extant [19]. In our study, treatment of mice with 1200 mg/kg PLX5622 resulted in ~ 93% reduction in the amount of microglia and ~ 80% of MGnD microglia. We note that MGnD microglia are conventionally regarded as activated by Aβ plaques, but seemingly can also become activated in response to AAV-P301L-tau injection as evaluated by MGnD markers, Clec7A and Mac2. Whether or not MGnD microglia elicited from these two pathologies are phenotypically different remains to be investigated in future studies.

### Microglia depletion and amyloid plaques

Microglia may play bidirectional roles for Aβ clearance via phagocytosis, endolysosomal clearance, and seeding of Aβ aggregates depending on the age of animals and the stage of Aβ accumulation in the brain. The general consensus of previous research regarding the effect of microglia depletion on amyloid deposition is that it is largely ineffectual aside from those utilizing the 5xFAD mouse model, which report dramatic improvements of Aβ pathology following microglia depletion [20, 47–49]. Here we found that microglia depletion causes a sizeable increase in compact, but not diffuse amyloid deposition (Supplemental Table S1). There are several possibilities which may explain the discrepancy between ours and the previous findings. Firstly, all of the previous studies assessing the effect of microglia depletion on amyloid deposition used transgenic models that overexpress human *APP* mutants (Supplementary Table S1). Deposition as a result of overexpression in combination with mutation may likely be more intense than the *App*^*NL-G-F*^ model, which replaces the intrinsic m*APP* gene with the human version expressing three mutations to increase amyloid deposition [24]. Therefore, depletion of microglia in the former case may not have as large an effect on amyloid deposition as in the latter; overexpression of *APP* may saturate the ability of microglia to clear Aβ, resulting in no effect of depletion. Furthermore, the heterogeneity of mouse models, onset, duration and efficiency of microglia depletion, and techniques for quantifying deposition all make comparisons difficult to interpret. Among them, only two reports from Spangenberg et al. [20, 21] and this study show achievement of at least 95% depletion of microglia and duration of at least 2 months.

These studies revealed a reduction in plaque size and number when microglia are depleted at a young age (1.5 months), suggesting that microglia may play a pathological role in the early stage of Aβ deposition. Most studies in which amyloid burden is assessed utilize 6E10 for staining, which recognizes residues 1–16 of Aβ. In this study, we find no effect of microglia depletion on amyloid deposition using the 82E1 monoclonal antibody, which recognizes the same residues. However, staining with 4G8, which recognizes residues 17–24 of Aβ, revealed a dramatic increase in diffuse plaque deposition that recapitulated the increases found in compact plaque deposition determined by thioflavin-S staining. We suspect the difference in plaque compaction is most visible through thioflavin-S staining compared to diffuse plaque staining possibly because diffuse portions of plaques are not rigid and fibrillar enough for microglia to interact with easily, and therefore not much difference is observed here. Future studies to investigate the impact of microglia depletion on Aβ should use a variety of markers and techniques to uncover results. Data presented here suggest that microglia are indeed highly involved in plaque compaction and clearance.

### Microglial depletion and NP tau

In this study, overexpression of mutant P301L-tau in the MEC of *App*^*NL-G-F*^ mice produced two different kinds of tau pathology: increased AT8^+^ neurons at the GCL and NP tau on amyloid plaques. Aggregated and phosphorylated tau in dystrophic neurites, recently described as NP tau, is a known pathological hallmark of AD brains [10, 13]. We observed an increase in NP tau accumulation by over-expressing P301L-tau mutation in the MEC of *App*^*NL-G-F*^ mice. This is supported by a recent study showing that tau fibril injection in *App*^*NL-F*^ mice induced NP tau accumulation [10]. We found that microglia depletion increased NP tau accumulation. Loss of Aβ compaction after microglial depletion could facilitate a larger volume of Aβ fibril-induced dystrophic neurites. This is consistent with the recent report demonstrating enhanced NP tau formation in APPPS1–12 mice with TREM2 knockout or R47H mutant background, which showed a diminished number of plaque-associated microglia [13]. Authors speculated that microglial dysfunction increases the susceptibility of dystrophic neurites to develop this pathology, possibly through increased vulnerability to the toxic effects of Aβ42 in plaques and not due to the reduction of the phagocytosis activity of plaque-associated microglia. Here, our results showing p-tau entrapped by plaque-associated MGnD microglia provide an additional interpretation: NP tau may be actively phagocytosed by plaque-associated MGnD and their depletion allows for greater accumulation of NP tau pathology. Thus, we speculate that phagocytosed NP tau may also be an important source for microglia to secrete tau seed-containing EVs.

### MGnD microglia-derived EVs and tau propagation

**Fig. 6:**
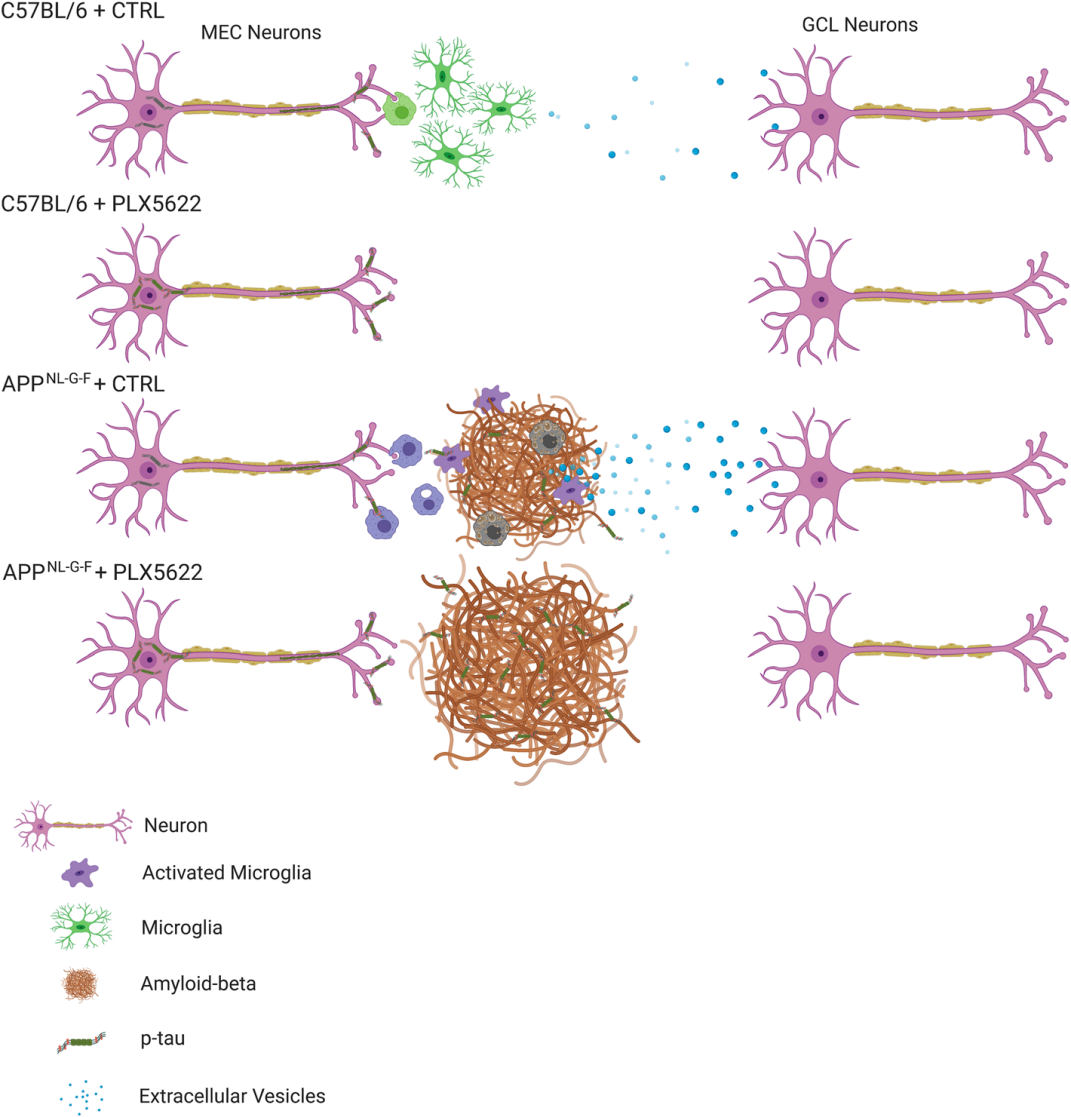
Schematic diagram of Aβ plaque deposition and microglia-mediated tau propagation. In the WT mouse brain, tau propagation from the MEC to GCL neurons is sensitive to microglia. In *App*^*NL-G-F*^ mouse brains, Aβ plaque associated microglia are more phagocytic and hyper-secrete tau-seeding EVs compared to homeostatic microglia, resulting in enhanced tau propagation from MEC to GCL regions. Both Aβ plaque and NP tau pathology are increased following microglia depletion, suggesting their active roles on compaction and clearance of Aβ plaques and NP tau

In addition to the new possibility of NP tau-mediated spread of tau pathology, microglia also phagocytose tau-containing synapses. It is well known that microglia actively phagocytose synapses by synaptic tagging with complements (C1q and C3) [50–52], which also play an important role in Aβ-induced synaptic loss [53]. As shown in the current and previous studies [36, 54], C1q signal is highly intense in the OML, and this study shows that it is microglia-dependent. C1q is likely to play a role in engulfing damaged synapses containing pathological tau seeds by microglia in the OML. Given that microglia are known to be activated and recruited to the plaque region where they surround, phagocytose, and also secrete toxic aggregates through their EVs, we hypothesize that the activity of plaque-associated MGnD microglia in this region may inadvertently bolster tau propagation from the MEC to the DG in *App*^*NL-G-F*^ mice (Fig. 6). Indeed, microglia-derived EVs are endocytosed by proximal neurons and influence their activity [55–57]. Another recent report showed that microglia isolated from AD brains or tau mice can seed tau in vitro [58]. We developed a way to measure EV release specifically from microglia in vivo using the mE-CD9 lentivirus. This allowed us to quantify the number of mE-CD9^+^ particles localized around infected microglia using super-resolution microscopy. We found that EV release was over three-times higher from MGnD microglia compared to homeostatic microglia. These data are supported by previous evidence showing that many EV markers, such as CD63, CD9, CD81, and Tsg101 are upregulated in MGnD microglia isolated from APP or APP/PS1 mice [8, 59]. We recapitulated this finding via qPCR using isolated Clec7A^+/−^ microglia and by immunofluorescence showing co-localization of Tsg101 and Clec7A in MGnD microglia around Aβ plaques in this study. We utilized the mE-CD9 lentivirus and AAV-P301L-tau to directly examine the interaction of mE-CD9^+^ microglia with p-tau^+^ neurons, which also induced MGnD microglia and appear to hyper-secrete EVs. Importantly, mE-CD9^+^ EVs contained p-tau, suggesting that microglia-derived EVs have the potential to seed p-tau aggregates in neighboring or distant cells, exacerbating tau propagation.

In summary, we provide strong evidence that MGnD microglia release dramatically higher levels of EVs in response to amyloid and tau pathology compared to inactivated microglia. Additionally, these EVs appear to contain pathologic p-tau, suggesting a potential mechanism explaining how Aβ plaques enhance propagation of tau pathology. It is possible that microglia depletion can alleviate the propagation of pathologic tau through alternative mechanisms, such as preventing microglia-mediated activation of tau kinases through inflammasomes [60–63]. Therefore, in order to specifically test the role of microglia-derived EVs in tau pathology propagation, future studies involving microglia-specific ablation of EV secretion are necessary.

## Methods

### Animals and genotyping

All mouse care and experimental procedures were approved by Institutional Animal Care and Use Committee of the Boston University School of Medicine. *App*^*NL-G-F*^ mice were bred and genotyped in-house. C57BL/6 were purchased from the NIA. Mice were caged in accordance with their own sex and housed in a barrier facility with 12 h light and 12 h dark cycles. Roughly half of the animals used were male and half female between WT and *App*^*NL-G-F*^ groups. Food and water was provided ad libitum. Throughout the life of all mice, veterinary staff closely monitored animals for complications. Genotyping for animals was conducted in house via PCR using the following primers [24]: E16WT: 5′ – ATCTCGGAAGTGAAGATG – 3′ E16MT: ATCTCGGAAGTGAATCTA WT: 5′ – TGTAGATGAGAACTTAAC – 3′ loxP: 5′ – CGTATAATGTATGCTATACGAAG – 3′. C1Q KO mice were obtained in good condition from Jackson Laboratories (Stock No: 031675).

### Viral vector production

The AAV2/6-SYN1-P301L tau (AAV-P301L-tau) was generated as previously described [22]. This virus is recombinant AAV2/6 (AAV2/6) pseudoserotype, and was created at Viral Core Facility, Boston Children’s Hospital. The vector consists of AAV-2 inverted terminal repeats, the human synapsin-1 gene promoter driving expression of human P301L tau 1–441, the woodchuck hepatitis virus post-transcriptional control element (*WPRE*) and a bovine growth hormone polyadenylation site. Iodixanol step gradient ultracentrifugation followed by heparin FPLC affinity chromatography and dialysis in PBS overnight was used to purify and prepare AAV particles. Viral titers were calculated via q-PCR. Purity of AAV was determined by SDS-PAGE and Coomassie brilliant blue staining.

### CSF1R inhibitor treatment and intracranial injection

PLX5622 or control (Plexxikon, Inc., San Francisco, CA), was impregnated into rodent chow at 1200 ppm (AIN-76A, Research Diet, Inc., Brunswick, NJ) and provided the animals in equal quantity starting at 4 months of age. After one month since the beginning of PLX5622 treatment, intracranial injections of AAV-P301L-tau were administered to the control- and drug-treated mice. This AAV expresses the mutant version of human tau P301L under the syn-1 promoter, which is neuron-specific [64]. Nine tenths of 1 μL were injected into each mouse with coordinates (AP: 4.75, ML: 2.90, DV: 4.64) at a viral titer of 1.2 × 10^11^ using a robotic stereotaxic drill & microinjection machine (Neurostar, Tubingen, Germany) attached with 10-μl syringe (Hamilton, model 701 LT, #80301) and glass capillary (Neurostar) held together with microelectrode holder (World Precision Instruments #MPH6S10). Mice were anesthetized during the procedure with 3% isoflurane and received 1 mg/kg meloxicam for pain relief. Experimenters were blinded to which group received PLX5622 and which received placebo throughout the experiment. Animals experiencing undue trauma or distress following injections were sacrificed and excluded from the study. Following one-month incubation of virus with continuous PLX5622 treatment, animals were sacrificed via transcardial perfusion with PBS and followed with fixation by 4% paraformaldehyde (PFA) solution. Brains were immediately harvested.

### Histological processing and immunofluorescence staining

Brains were immersed in 4% PFA at 4C° for overnight following harvest. The following day, brains were placed in 30% sucrose solution in PBS in 4C° for overnight again in preparation for cryosectioning. Sagittal brain slices were then gathered using a Cryostar NX50 (Thermo Fisher Scientific, # 957250 K) at 30 μm thickness. Following sectioning, brains were mounted on superfrost plus microscope slides (Thermo Fisher Scientific, #22–037-246) and stored at − 80 °C. The following antibodies and reagents were used for immunofluorescence staining: 4G8 1:100 (Aβ_17–24_, BioLegend, #800704), AT8 1:300 (pSer^202^/pSer^205^ tau, Thermo, #MN1000), GFAP 1:300 (Cell Signaling Bio, #36705), C1q 1:300 (Abcam, #ab182451), 82E1 1:100 (Aβ1–16, IBL, #10323), thioflavin-S 1:50 in 50% ethanol (Sigma, #T1892-25G), 1-Fluoro-2,5-bis[(E)-3-carboxy-4-hydroxystyryl] benzene (FSB, 50 μM) in 10% ethanol (Millipore Sigma, #07602), HT7 1:300 (human tau, Thermo, #MN1000), Clec7A 1:100 (InvivoGen, #mabg-mdect), Tsg101 1:300 (Santa Cruz Biotech, #sc-7964), IBA1 (Wako, #019–19,741), DAPI 1:2500 (Thermo Fisher Scientific, #62248). Rabbit polyclonal anti-mouse P2RY12 antibody 1:600 was generously provided by Dr. Oleg Butovsky’s laboratory [65, 66]. Sections were washed with PBS prior to antigen retrieval with 88% formic acid except for staining with anti-GFP antibody (Santa Cruz Biotech, #sc-101,536). Blocking solution consisted of 5% normal goat or donkey serum, 5% bovine serum albumin (BSA), and 1% Triton X-100. All following primary and secondary staining buffer consisted of 5% BSA, 1% Triton X-100 in PBS. Following staining, sections were allowed to air-dry and then mounted using Fluoromount-G (Invitrogen, #00–4958-02). Tiled images were taken at 20X with an Eclipse Ti microscope (Nikon Instruments). Confocal images were taken using SP8 laser confocal microscope with Lightning (Leica). Images were observed and analyzed using open-source image processing package FIJI. Processing of z-stack images and quantification of mE-CD9^+^ particles was accomplished using IMARIS rendering software (Oxford Instruments). The face of each z-stack is 50-μm by 50-μm and the thickness is approximately 30 μm.

### Microglia isolation and sorting

Mice were transcardially perfused with ice-cold Hanks Balanced Salt Solution (HBSS) and whole brains were removed. Brain tissue was dissected into 1-mm^3^ pieces and homogenized into a single cell solution in ice cold HBSS using a glass Dounce homogenizer. Single cell suspensions were centrifuged over a 37%/70% discontinuous Percoll (GE Healthcare) gradient. Mononuclear cells were isolated from the interphase of these layers. In order to distinguish resident microglia from recruited myeloid cells, we used a monoclonal antibody that recognizes FCRLS, which is expressed on microglia, but not infiltrating myeloid cells [8, 65]. Isolated cells were stained with anti-FCRLS-APC, along with CD11b-PeCy7, Ly6C-PercpCy5.5, LIVE/DEAD Blue Dead Cell Stain for UV Excitation, followed by rat primary antibody against Clec7A (1:100) (InvivoGen) and secondary anti-rat FITC. Doublets were removed and the LIVE/DEAD^LO^ CD11b^HI^ Ly6C^LO^ FCRLS^+^ Clec7A^+/−^ microglia populations were isolated.

### RNA isolation, cDNA synthesis, and quantitative PCR

For purification of microRNA and total RNA from isolated microglia, the miRNeasy Mini Kit was used according to manufacturer protocols (Qiagen, #217004). cDNA synthesis was achieved using the Superscript VILO cDNA synthesis kit (Thermo Fisher Scientific #11754050) with an initial RNA input of 100 ng after DNase I digestion (Ambion, # AM2222). Quantitative PCR was carried out in 6 μL total volume. Each reaction volume consisted of 0.5 μL of primers (Thermo Fischer Scientific, # 4331182), 1.5 μL of cDNA, 3 μL of water, and 1 μL of Taqman Fast Advanced Master Mix (Thermo Fisher Scientific, #4444557). The amplification was conducted using an ABI Prism 7900HT Sequence Detection System.

### Lentiviral vector production

The pLV-EF1α-mEmerald-CD9-miR9T lentivirus was generated by modifying commercially available pLV.PGK.GFP.miR9T lentivirus [39]. The vector backbone was modified to contain the murine EF1α promoter and express mEmerald conjugated to CD9 followed by miR9T in the 3’UTR. mEmerald-CD9–10 was a gift from Michael Davidson (Addgene plasmid # 54029; http://n2t.net/addgene:54029; RRID:Addgene_54,029). Viral particle production and packaging was done by a commercial source (SignaGen Laboratories, Rockville MD, USA). The viral titer is >1E + 9 TU/ml. Lentivirus (1 μL) was injected to the MEC (AP: 4.75, ML: 2.90, DV: 4.64) of 6 months-old *App*^*NL-G-F*^ and WT mice and euthanized at 10-day post-injection for immunohistochemical and biochemical analyses.

### Cell culture transduction

Human embryonic kidney 293 T (HEK293T) cells were cultured in 24-well tissue culture-treated plates with 1% penicillin-streptomycin (Thermo Fischer Scientific, #15140122), 10% fetal bovine serum (FBS, Thermo Fischer Scientific, #A3382001) in Dulbecco’s Modified Eagle Medium (DMEM, Invitrogen #11965118) for 24 h prior to plasmid DNA transduction. HEK293T cells were serum-starved for 1 h in DMEM prior to receiving 1 μL of mE-CD9 lentivirus or control lentivirus.

### Immunoblotting

Transfection was performed with pLV-EF1α-GFP-miR9T or pLV-EF1α-mEmerald-CD9-miR9T plasmid (2.5 μg/well) using Lipofectamine 3000 (Thermo Fisher Science, #L3000008). Whole cells and conditioned medium were collected 3 d after transfection. The conditioned medium was centrifuged at 2000 *g* for 30 min, at 10,000 *g* for 30 min, at 144,000 *g* for 70 min and last pellets were collected as the EV fraction. Whole cells or EV fractions were lysed with RIPA buffer containing Halt Protease and Phosphatase inhibitor Cocktail (Thermo Fisher Science, #78442), and incubated at 95 °C for 5 min with Laemmli sampling buffer (Bio-Rad, #S3401-10VL) supplemented with 2% v/v 2-mercaptoethanol. The protein samples (10 μg/lane) were subjected to 10% SDS-PAGE, transferred to PDVF membrane (Bio-Rad), and blocked with 3% skim milk in TBS 0.5% Tween20 (TBS-T). The membranes were incubated overnight at 4 °C with anti-GFP mouse monoclonal antibody (Santa Cruz Biotech, B-2; 1:500), and washed with TBS-T. Then the membranes were incubated with HRP-conjugated anti-mouse IgG antibody (Cell Signaling Technology, #7076; 1:10,000) for 1 h at room temperature. The proteins were detected with Immobilon chemiluminescent HRP substrate (Millipore Sigma, #WBKLS0100) and visualized with Chemiluminescent Western Blot Imaging System (Azure, #C300).

### Purification of EVs

Following sacrifice and transcardial perfusion with cold PBS, mouse brains were cut in half sagittally and the left half was used for EV purification. Left hemispheres were sliced with a razor into thin strips > 1 mm in width and subjected to digestion in 1.5 mL of collagenase diluted into Hibernate E (Invitrogen, #A1247601) (75 U/mL) (Worthington Biochemical, #LS004180) at 37 degrees Celsius for 15 min with occasional stirring. Next, protease inhibitor was added at the recommended dilution (Thermo Fisher, #P178443) and the brains were mechanically homogenized for approximately 2 min and then subjected to 40-μm filtration (Fisher Scientific, #22–363-547). Solutions were then processed by sequential centrifugation in the following order and the supernatant was used for the next step each time: 300 *g* for 10 min, 2000 *g* for 10 min, and 10,000 *g* for 10 min. Solutions were then filtered through 0.22-μm filters and subjected to ultracentrifugation at 140,000 *g* for 70 min. Lastly, pellets were subjected to sucrose gradient and separated at 200,000 *g* for 20 h. The 0.65 M and 0.8 M sucrose fractions were pooled then diluted in 12 ml of cold PBS and subjected to ultracentrifugation at 140,000 *g* for 70 min.

### Immunoelectron microscopy

The EV pellet was then fixed in 4% formaldehyde with 0.1% glutaraldehyde for 2 h at RT then washed with PBS containing 20 mM glycine. Following fixation, the pellet was processed for ultra-thin cryosectioning. The pellets were infiltrated with 2.3 M sucrose in PBS for 15 min, frozen in liquid nitrogen, and then sectioned 80-nm thick at -120C° as previously described [22]. Sections were transferred to a carbon-coated copper formvar grid and labelling was conducted on a piece of parafilm. Blocking with 1% BSA for 10 min was used to prevent non-specific labelling. All antibodies were diluted in 1% BSA in PBS. Labeling for p-tau was conducted using AT8 1:30 (pSer202/pSer205 tau, Thermo Fisher Scientific, #MN1000) for 30 min, followed by rabbit anti-mouse bridging antibody (1:50, AbCam, ab6709) for 30 min and 5-nm protein A-gold (1:50, University Medical Center, Utrecht, the Netherlands) for 20 min. After four washes in PBS, the grids were fixed for 5 min in 1% glutaraldehyde in PBS, followed by four washes in 20 mM glycine in PBS (to quench free aldehyde groups). mEmerald-CD9 was labelled using anti-GFP rabbit polyclonal antibody (AbCam ab6556; 1:30) followed by 10-nm Protein A gold (1:50). This was followed by washing with PBS and water for 15 min. Sections were then contrasted with 0.3% uranyl acetate in 2% methylcellulose for 5 min. Grids were examined at 80 kV with a TecnaiG2 Spirit BioTWIN transmission electron microscope. Images were recorded with an AMT 2 k CCD camera.

### Statistical analysis

All statistical analyses were performed in GraphPad Prism 8 (Graph-Pad Software, Inc). Two-way ANOVA was used to assess comparisons between all four experimental groups when applicable. Normal distributions were assumed when making post-hoc analyses and correcting for multiple comparisons (Tukey). In instances of two group comparisons, unpaired t-tests assuming equal variances were used.

## Supplementary Information

**Additional file 1: Supplementary Table S1.** Studies of microglial depletion on AD mouse models. **Supplementary Table S2.** Studies of microglial depletion on mouse models of tauopathy. **Fig. S1.** Effects of microglia depletion on plaque deposition and astrogliosis in *App*^*NL-G-F*^ mice. **Fig. S2.** Assessment of tau propagation in AAVP301L-tau injected WT and *App*^*NL-G-F*^ mice. **Fig. S3.** Gating strategy for the FACS isolation of Cd11b^hi^ Ly6c^lo^ Fcrls^+^ Clec7A^±^ microglia from aged *App*^*NL-G-F*^ mice. **Fig. S4.** Characterization of mE-CD9 lentiviral vector in vitro and in vivo. **Fig. S5.** MGnD induction in WT vs *App*^*NL-G-F*^ brains.

## Data Availability

Data produced and analyzed in this study are all available herein. Datasets used for analysis are available from the corresponding author upon reasonable request.

## Abbreviations

AAV: adeno-associated virus
Aβ: amyloid-β peptide
AD: Alzheimer’s disease
APOE: apolipoprotein E
APP: amyloid precursor protein
C1q: complement component 1q
CA: *Cornu Ammonis*
CD11b: cluster of differentiation molecule 11b/ integrin alpha M
Clec7A: C-type lectin domain family 7, member A
CSF1R: colony stimulating factor 1 receptor
DG: dentate gyrus
EVs: extracellular vesicles
FACS: Fluorescence-activated cell sorting
FCRLS: Fc receptor-like S, scavenger receptor
FSB: 1-Fluoro-2,5-bis[(E)-3-carboxy-4-hydroxystyryl]benzene
GCL: granular cell layer
GFAP: glial fibrillary acidic protein
HEK293: human embryonic kidney cells 293
Ly6c: lymphocyte antigen 6 complex, locus C
mE-CD9: mEmerald-CD9 fusion protein
MGnD: neurodegenerative microglia
MEC: medial entorhinal cortex
mi9RT: tandem miR-9 target sequence
NFT: neurofibrillary tangle
NP: neuritic plaque
OML: outer molecular layer
PFA: paraformaldehyde
p-tau: phosphorylated tau
TA: temporoammonic
TREM2: triggering receptor expressed on myeloid cells 2
Tsg101: tumor susceptibility gene 101
WB: western blot
WT: wild type
