# Correction to: Plaque associated microglia hyper-secrete extracellular vesicles and accelerate tau propagation in a humanized APP mouse model

**DOI:** 10.1186/s13024-021-00447-2

**Published:** 2021-04-14

**Authors:** Kevin Clayton, Jean Christophe Delpech, Shawn Herron, Naotoshi Iwahara, Maria Ericsson, Takashi Saito, Takaomi C. Saido, Seiko Ikezu, Tsuneya Ikezu

**Affiliations:** 1 Departments of Pharmacology and Experimental Therapeutics, Boston University School of Medicine, Boston, MA 02118 USA; 2 Department of Cell Biology, Harvard Medical School, Boston, MA 02115 USA; 3 Department of Neurocognitive Science, Institute of Brain Science, Nagoya City University Graduate School of Medical Sciences, Nagoya, Aichi Japan; 4 Laboratory for Proteolytic Neuroscience, RIKEN Center for Brain Science, Wako, Saitama Japan; 5 Center for Systems Neuroscience, Boston University, Boston, MA 02215 USA; 6 Department of Neuroscience, Mayo Clinic Florida, Jacksonville, FL 32224 USA

**Correction to: Mol Neurodegeneration 16, 18 (2021)**

**https://doi.org/10.1186/s13024-021-00440-9**

The original article [1] contained an error whereby the captions for Additional Files 2 and 3 were mistakenly inverted. This has since been corrected.

## References

[1] Clayton K (2021). Plaque associated microglia hyper-secrete extracellular vesicles and accelerate tau propagation in a humanized APP mouse model. Mol Neurodegeneration..

