# Supplementary material for: Plaque associated microglia hyper-secrete extracellular vesicles and accelerate tau propagation in a humanized APP mouse model
Source: Mol Neurodegener. 2021 Mar 22;16:18. doi: 10.1186/s13024-021-00440-9 (PMC7986521; doi:10.1186/s13024-021-00440-9)
Supplement: Supplementary file 4 — Additional file 1: Supplementary Table S1. Studies of microglial depletion on AD mouse models. Supplementary Table S2. Studies of microglial depletion on mouse models of tauopathy. Fig. S1. Effects of microglia depletion on plaque deposition and astrogliosis in AppNL-G-F mice. Fig. S2. Assessment of tau propagation in AAVP301L-tau injected WT and AppNL-G-F mice. Fig. S3. Gating strategy for the FACS isolation of Cd11bhi Ly6clo Fcrls+ Clec7A± microglia from aged AppNL-G-F mice. Fig. S4. Characterization of mE-CD9 lentiviral vector in vitro and in vivo. Fig. S5. MGnD induction in WT vs AppNL-G-F brains. [file 13024_2021_440_MOESM1_ESM.pdf]

Supplementary information

Plaque associated microglia hypersecrete extracellular vesicles while clearing the pathological proteins in a humanized APP mouse model

Microglia Depletion and Amyloid Deposition										
Study	Model	Onset of Plaque Deposition	Depletion Method	% Depletion and Method	Age at Depletion	Depletion Duration	Plaque Size	Plaque Number	Aβ Concentration	Region(s) of Interest
Spangenberg 2019 [20]	5XFAD	1.5 months	PLX5622 (1200 ppm)<br>PLX3397 (600 ppm)	~99% (Iba1 staining)<br>~99% (Iba1 staining)	1.5 months	10 and 24 weeks	-35% (Thioflavin S)	-60% (Thioflavin S, 6E10 staining)	No change (ELISA)	Cortex
Casali 2020 [58]	5XFAD	1.5 months	PLX5562 (1200 mg/kg)	30-70%% (Iba1 staining)	4 months	4 weeks	-	-~30% (Thioflavin S)<br>+~35% (6E10 staining)	-	Cortex, Hippocampus<br>Subiculum,<br>Thalamus
Son 2020 [59]	5XFAD	1.5 months	PLX3397 (50 mg/kg)	~40% (Iba1 WB)	9 months	1 month	-	-	-33% (WB)	Cortex, Hippocampus
Sosna 2018 [60]	5XFAD	1.5 months	PLX3397 (290 mg/kg)	~70-80% (Iba1 staining)	2 months	3 months	-40% (6E10 staining)	-90% (6E10 staining)	~90% (Dot Blot)	Cortex, Hippocampus
Gratwohl 2009 [17]	APPPS1	1.5 months	CD11b-HSVTK (Ganciclovir pump 50 mg/ml)	>90% (Iba1 staining)	1.5 months<br>3 months<br>5 months	3 weeks<br>4 weeks	No change (Congo red staining)	No changes (Congo red staining)	No change (WB, ELISA)	Cortex
Dagher 2015 [19]	3xTg-AD	6 months	PLX5622 (300 mg/ kg)	30% (Iba1 staining)	2 months	6 weeks<br>13 weeks	No change (Thioflavin S staining)	No changes (Thioflavin S)	No changes (ELISA)	Subiculum
Zhong 2019 [61]	5xFAD	1.5 months	PLX3397 (290 mg/kg)	75% (Iba1 staining)	7 months	3 weeks	No change (MOAB-2 staining)	No change (MOAB-2 staining)	-	Hippocampus
Alonso 2016 [62]	APPPS1	1.5 months	GW2580 (75 mg/kg)	50% (PU.1 & Iba1 staining)	9 months	3 months	No change (6E10 staining)	No change (6E10 staining)	No changes (ELISA)	Cortex
Spagenberg 2016 [21]	5xFAD	1.5 months	PLX3397 (600 mg/kg)<br>PLX5622 (1200 mg/kg)	80% (Iba1 staining)<br>95% (Iba1 staining)	10 months<br>1.5 months<br>14 months	4 weeks<br>4 weeks	No change (6E10, Thioflavin S staining)	No change (6E10, Thioflavin S staining)	No changes (ELISA)	Hippocampus , Cortex, Thalamus
Zhao 2017 [18]	APPSwe/PSEN1dE9 x CX3CR1Cre/+ Rosa26 iDTR	6 months	Diptheria toxin	~100% (Iba1 staining)	>12 months	2 weeks	+13% (Congo red staining)	No change (Congo red staining)	-	Cortex
Clayton 2021	*APPNL-G-F*	2 months	PLX5562 (1200 mg/kg)	95% (Iba1 + P2RY12 staining)	4 months	2 months	+30% (Thioflavin S staining)<br>+69% (4G8 staining)<br>No changes (82E1 staining)	+50% (Thioflavin S staining)<br>No changes (82E1 staining)	-	Cortex

Supplementary Table S1. Studies of microglial depletion on AD mouse models.
Green rows: Studies showing reduction in Aβ deposition; Yellow-green rows: Studies showing no changes in Aβ deposition; Beige rows: Studies showing enhanced Aβ deposition.

Microglia Depletion and Tau Accumulation									
Study	Model	Onset of Tau Pathology	Depletion Method	% Depletion	Age at Depletion	Depletion Duration	Tau Accumulation	Degeneration	Region(s) of Interest
Bennett 2018 [1]	Tg4510	2.5 months	PLX3397 (290 mg/kg)	30% (Iba1 staining)	12 months	3 months	No changes (FRET, hTau ELISA, AT8 WB, pT231 WB, pP8 WB)	-	Whole Brain (biochemistry)<br>Cortex (histology)
Mancuso 2019 [2]	PS19	3 months	JNJ-527<br>(30 mg/kg)	40% (CD11b/CD45 FACS)	8 weeks	8 weeks	-~66% (AT8/Total Tau WB)<br>~-75% (Insoluble/Total Tau WB)	~-25% motor neuron loss (Nissl staining)<br>~+15% latency to fall (rotarod test)	Whole Brain (biochemistry)<br>Spinal Cord (histology)
Shi 2019 [3]	PS19 x hApoE4<br>PS19 x ApoE KO	3 months	PLX3397 (400 mg/kg)	~100% (Iba1 staining)	6 months	3.5 months	~-75% (AT8+ area)<br>-50-66% (Insoluble pTau and hTau ELISA)	~100% reversal hippocampal volume loss<br>~100% reversal cortical volume loss	Cortex (biochemistry)<br>Hippocampus (histology)<br>Cortex (histology)
Zhu 2019 [4]	hTau x $Cx3cr1^{CreER}$ $R26^{DTA}$	9 months	Tamoxifen (400 mg/kg chow)	~50% (F4/80 FACS)	7-16 months	3 months	No changes (soluble Tau ELISA, insoluble Tau ELISA, CP13 staining)	-	Forebrain (biochemistry)<br>Cortex (histology)<br>Hippocampus (histology)<br>Cerebellum (histology)
Asai 2015 [5]	C57BL/6<br>PS19	3 months	PLX3397 (290 ppm)	~85% (Iba1 staining)	4 months	2 months	~-66% (AT8 staining)<br>~-50% (AT8 propagation)	~100% reversal spike amplitude loss	Hippocampus (histology)<br>Cortez (histology)<br>Hippocampus (electrophysiology)

Supplementary Table S2. Studies of microglial depletion on mouse models of tauopathy.

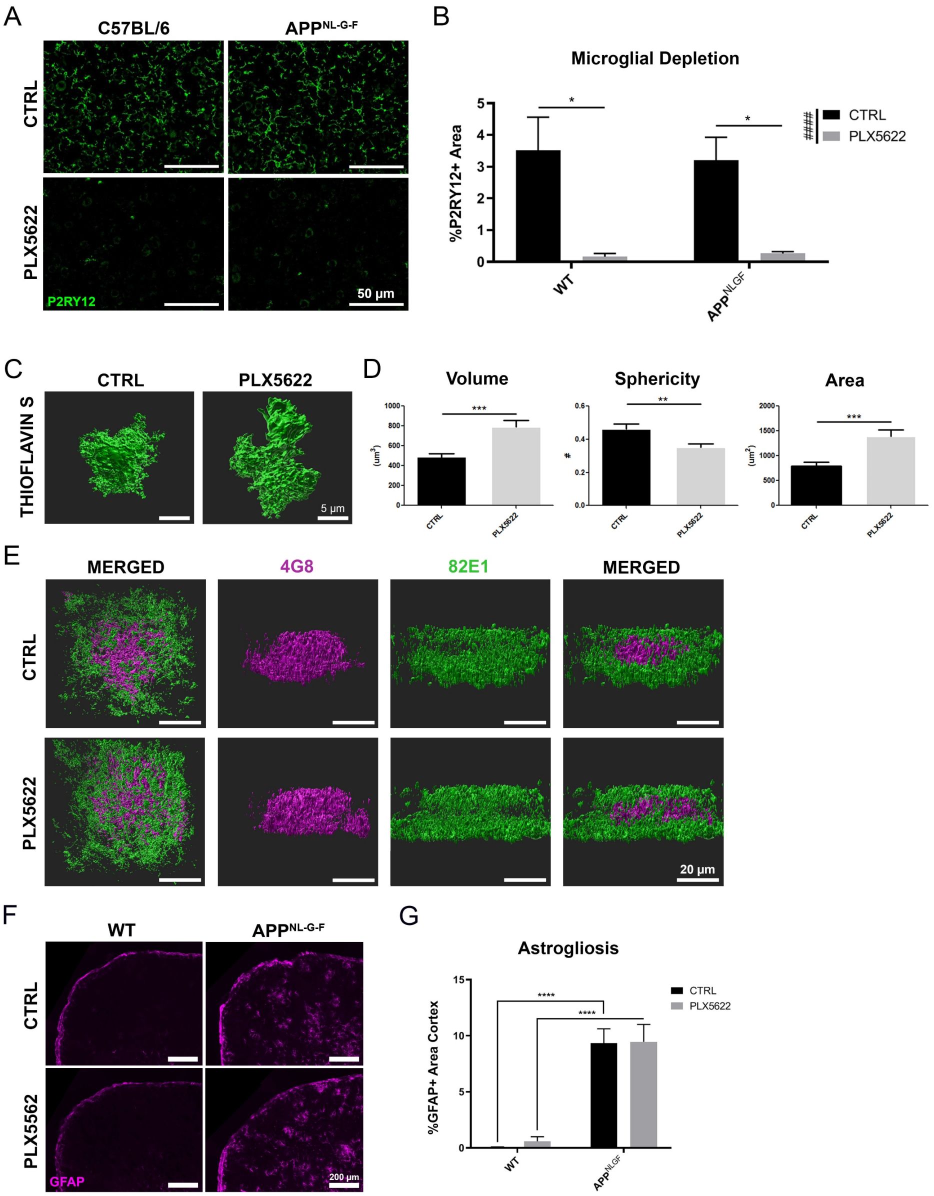

Fig. S1 Effects of microglia depletion on plaque deposition and astrogliosis in *App*$^{NL\text{-}G\text{-}F}$ mice. A. Representative images of P2RY12 staining in the cortical region from WT and *App*$^{NL\text{-}G\text{-}F}$ mice administered with control (CTRL) or PLX5622 chow. **B.** Unbiased quantification of percentage P2RY12^{+} area in the cortex**. C.** Representative 3D rendering of Thioflavin S^{+} plaques with and without microglia depletion. **D.** Unbiased quantification of plaque volume, sphericity, and overall area (n = 20 plaques per group). **E.** Representative 3D rendering of 4G8^{+} and 82E1^{+} plaques showing distinct staining patterns. **F.** Representative images of GFAP staining in the cortex. **G.** Unbiased quantification of GFAP^{+} area in the cortex. Representative images displayed in **A-G** are a mix of male and female mice. All values displayed represent the mean ± SEM. Graphs comparing values across all 4 groups were analyzed via 2-way ANOVA with Tukey post-hoc analysis for individual comparisons. $^{**}p < 0.01$, $^{***}p < 0.001$, $^{****}p < 0.0001$, #### $p < 0.0001$ for PLX5622 factor.

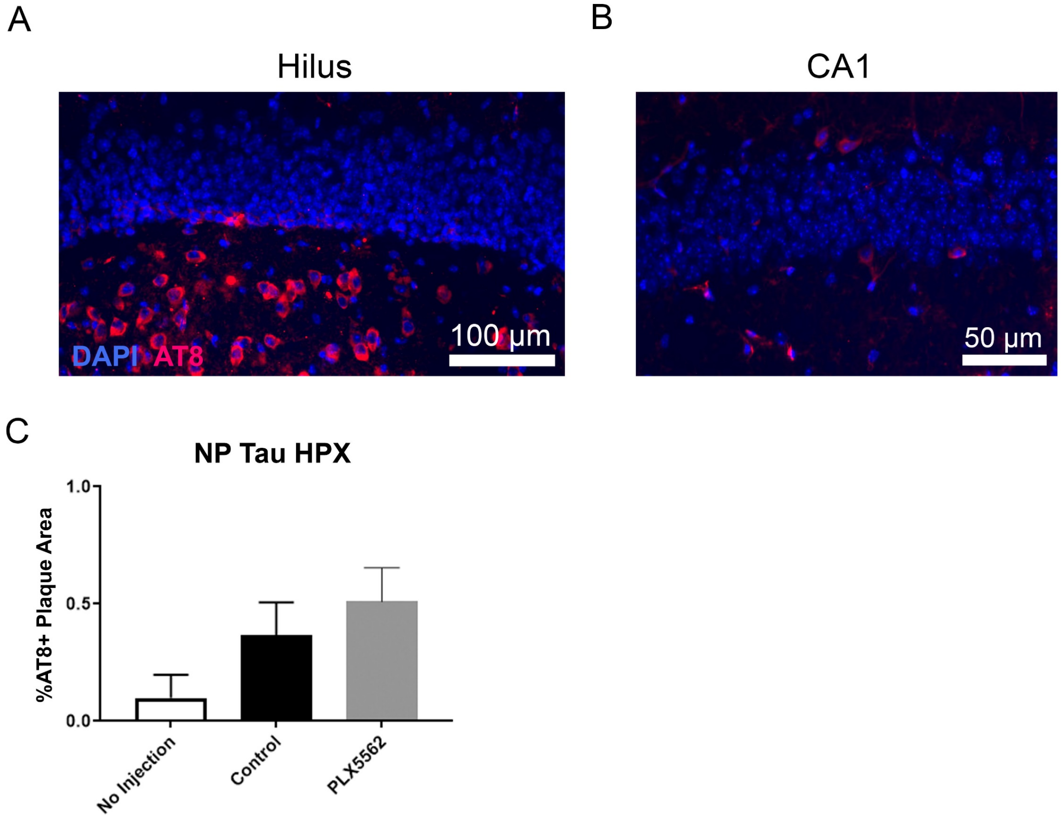

Fig. S2 Assessment of tau propagation in AAV-P301L-tau injected WT and *App*$^{NL\text{-}G\text{-}F}$ mice. **A.** Representative picture of strong tau propagation to the Hilus region of the hippocampus of *App*$^{NL\text{-}G\text{-}F}$ mice (AT8; red, DAPI; blue). **B.** Representative image of tau propagation to the CA1 region of the hippocampus. **C.** Unbiased quantification of percentage AT8+ NP tau in plaque area in the whole hippocampal region of *App*$^{NL\text{-}G\text{-}F}$ mice. Representative images displayed in **A-B** are a mix of male and female mice. All values displayed in C represent the mean ± SEM for a minimum of 6 animals per group. The graph comparing values across all 3 groups was analyzed via 1-way analysis of variance (ANOVA) with Tukey post-hoc analysis for individual comparisons.

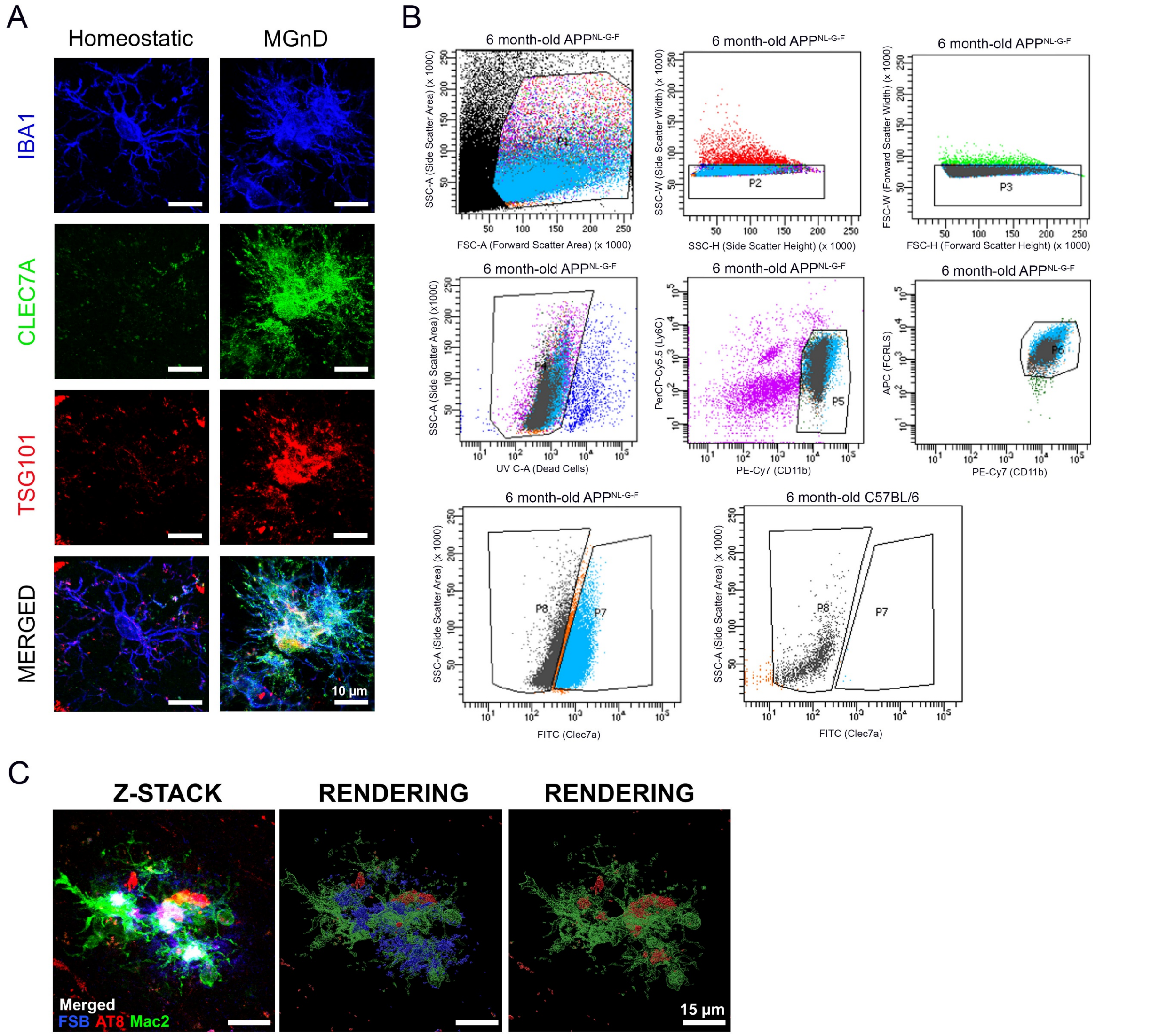

Fig. S3. Gating strategy for the FACS isolation of Cd11b^{hi} Ly6c^{lo} Fcrls^{+} Clec7A$^{\pm}$ microglia from aged *App^{NL-G-F}* mice. A. Representative high-magnification images of homeostatic microglia and MGnD in *App^{NL-G-F}* mice. Iba1 (blue), Clec7a (green), and Tsg101 (red). See also Supplemental Video S1 for the presence of Tsg101 within Clec7a^{+} microglia. **B.** Gating settings for fluorescence activated cell sorting of Clec7A$^{\pm}$ microglia from the brains of 6 months-old *App^{NL-G-F}* mice. Following live/dead cell and singlet selection, microglia were selected via a combination of Ly6C, CD11b, and FCLRS prior to being sorted as Clec7A^{+} or Clec7A^{-} microglia. P1: Forward/Side scatter screened cells, P2: Side scatter height/width screened cells, P3: Forward scatter height/width screened cells, P4: Dead cells/side scatter area screened cells, P5: Cd11b^{hi} Ly6c^{lo} cells, P6: Cd11b^{hi} Fcrls^{+} cells, P7: Clec7A^{+} cells, P8: Clec7A^{-} cells. Cells from C57BL/6 (WT) mice were used for defining the P7 and P8 fraction. **C.** Z-stack image and renderings of NP tau phagocytosed by MGnD in the MEC of *App^{NL-G-F}* mice. FSB (blue), AT8 (red) and Mac2 (green). See also Supplemental Video S2. Representative images displayed in **A-C** are a mix of male and female mice.

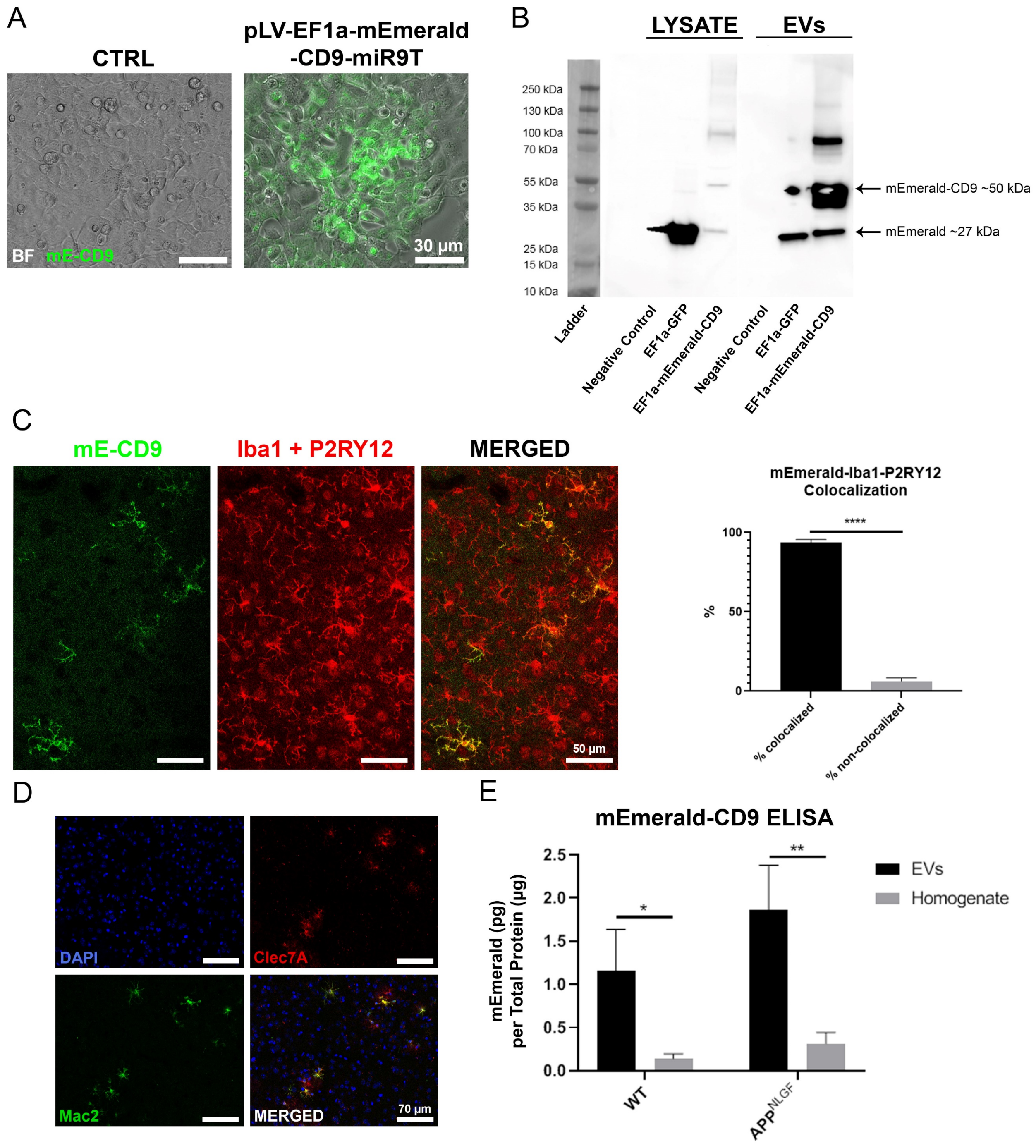
A
CTRL
pLV-EF1a-mEmerald
-CD9-miR9T
BF mE-CD9
30 µm
B
LYSATE
EVs
250 kDa
130 kDa
100 kDa
70 kDa
55 kDa
35 kDa
25 kDa
15 kDa
10 kDa
mEmerald-CD9 ~50 kDa
mEmerald ~27 kDa
Ladder
Negative Control
EF1a-GFP
EF1a-mEmerald-CD9
Negative Control
EF1a-GFP
EF1a-mEmerald-CD9
C
mE-CD9
Iba1 + P2RY12
MERGED
50 µm
mEmerald-Iba1-P2RY12
Colocalization
****
100
%
50
0
% colocalized
% non-colocalized
D
DAPI
Clec7A
Mac2
MERGED
70 µm
E
mEmerald-CD9 ELISA
mEmerald (pg)
per Total Protein (µg)
2.5
2.0
1.5
1.0
0.5
0.0
*
**
WT
APPNLGF
EVs
Homogenate

Fig. S4: Characterization of mE-CD9 lentiviral vector *in vitro* and *in vivo*. A. Representative images of mEmerald expression following control lentivirus (left) and mE-CD9 lentivirus (right) transduction in HEK293T cells following 48-hour incubation. **B.** Western blot of cell lysates and purified EVs from transiently-transfected HEK293T cells demonstrating expression of mEmerald-CD9 as determined by GFP blotting. **C.** Representative image and quantification of 10-day mE-CD9 lentivirus transduction into the MEC, demonstrating 94% colocalization of mE-CD9 (green) with microglial markers IBA1 and P2RY12 (both red). **D.** Representative images of overlap between Clec7A and Mac2 staining, demonstrating very similar populations of MGnD microglia in $App^{NL\text{-}G\text{-}F}$ mouse brain. **E.** Quantification of mE-CD9 in whole brain homogenate and extracellular vesicles isolated from mE-CD9 lentivirus-injected brains by GFP ELISA. Representative images displayed in **A-E** are a mix of male and female mice. All values displayed in C & E represent the mean ± standard error (SEM) for a minimum of 5 animals per group. Graphs comparing two groups were analyzed via Unpaired t-test. Graphs comparing values across all 4 groups were analyzed via 2-way analysis of variance (ANOVA) with Tukey post-hoc analysis for individual comparisons. * $p < 0.05$, ** $p < 0.01$, **** $p < 0.0001$, between indicated groups.

A

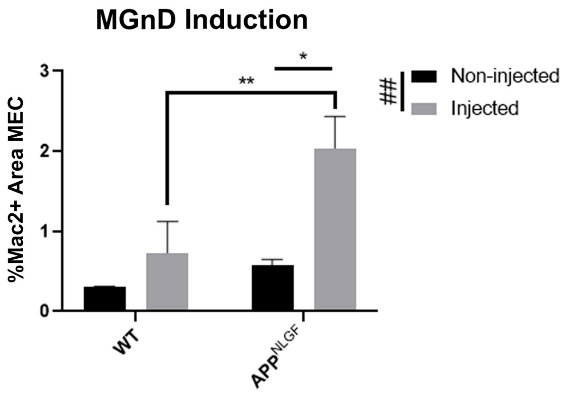

Fig. S5. MGnD induction in WT vs *App^NL-G-F* brains. The graph comparing values across all 4 groups were analyzed via 2-way analysis of variance (ANOVA) with Tukey post-hoc analysis for individual comparisons. All values displayed in A represent the mean ± SEM for a minimum of 5 animals per group. * $p < 0.05$ between indicated groups, ** $p < 0.01$ between indicated groups, ## $p < 0.01$ for the injection factor.

Supplementary Video S1: Phagocytosis of NP tau by Clec7a+ microglia.

FSB (Blue, Ab plaque)

AT8 (Red, NP tau)

Clec7a (Green, microglia)

Supplementary Video S2: Co-localization of Tsg101 in Clec7a+ microglia.

DAPI (Blue, nucleus)

Clec7a (Green, microglia)

Tsg101 (Magenta)

4G8 (Red, Ab plaque)

Supplementary Video S3: p-tau+ EV secretion from microglia.

DAPI (Blue, nuleus)

Mac-2 (Red, microglia)

GFP (Green / White, mE-CD9)

AT8 (Magenta, p-tau)
